# Developmental processes in the Rosaceae through the lens of DNA and RNA methylation

**DOI:** 10.1007/s00425-025-04623-2

**Published:** 2025-02-08

**Authors:** Tamunonengiye-Ofori Lawson, Juan-Pablo Selva, José Carballo, Mario Caccamo, Daniel J. Sargent, Nada Šurbanovski

**Affiliations:** 1 NIAB, 93 Lawrence Weaver Road, Cambridge, CB3 0LE UK; 2 Centro de Recursos Naturales Renovables de la Zona Semiárida (CERZOS), Universidad Nacional del Sur-Consejo Nacional de Investigaciones Científicas y Técnicas (CONICET), Bahía Blanca, Argentina; 3 Departamento de Biología, Bioquímica y Farmacia, Universidad Nacional del Sur (UNS), Bahía Blanca, Argentina

**Keywords:** DNA methylation, RNA methylation, Plant development, Rosaceae, Epigenetics, RdDM

## Abstract

**Main conclusion:**

This review discusses the DNA and RNA methylation pathways and their biological roles in Rosaceae developmental processes relevant for breeding and production.

**Abstract:**

The Rosaceae is a plant family of great importance for human nutrition and health. Many traits and developmental processes of the Rosaceae are influenced by epigenetic methylation, functions of which are now being unravelled in several important species of this family. Methylation of DNA at the 5th position of cytosine (5mC) is a well-established epigenetic mark that affects important cellular processes such as gene expression and genome stability and is involved in a wide range of plant biological functions. Further to this, recent technological advances have uncovered other naturally occurring chemical modifications of DNA and RNA as additional layers of regulatory epigenetic information in plants. In this review we give a comprehensive summary of plant 5-methylcytosine DNA methylation mechanisms and review their components identified in species of the Rosaceae family. We detail and discuss the role of 5mC DNA methylation dynamics in Rosaceae developmental processes, including phase transition, bud development, bud dormancy, plant architecture, plant regeneration, fruit development, ripening and senescence. We then review recent advances in understanding the newly identified nucleic acid modifications, *N*^*6*^-adenosine methylation of DNA (6mA) and RNA (m^6^A) as additional epigenetic mechanisms. We summarise identified components of adenosine methylation pathways in the Rosaceae and discuss the emerging roles of this modification in plant development including recent findings in Rosaceous species. Integrating epigenetic aspects of plant development with plant genetics and physiology is crucial for understanding biological processes in Rosaceous plants.

## Introduction

**Fig. 1:**
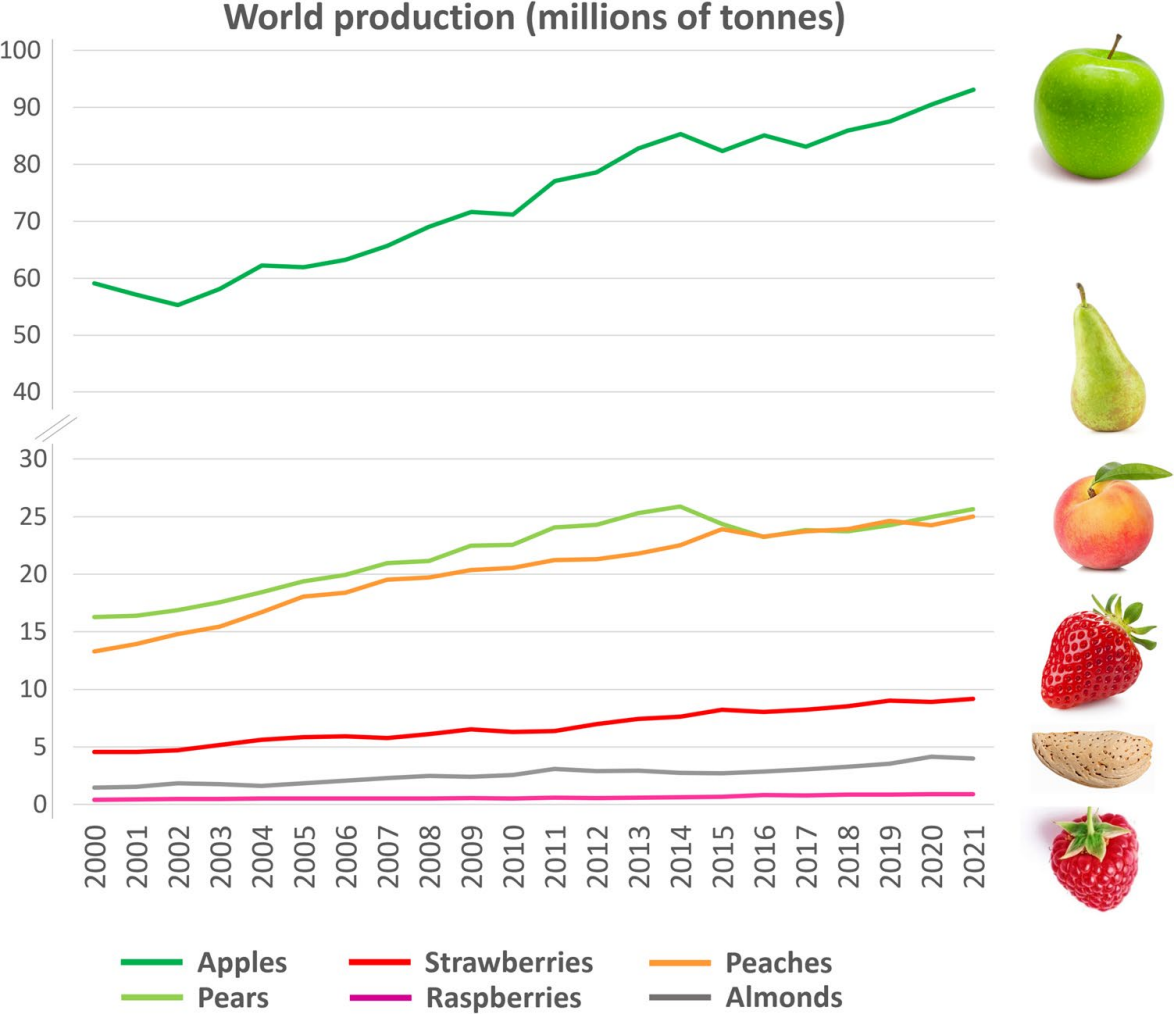
World production of major Rosaceous crops. Production of Rosaceous crops has been increasing for the last 20 years with apple being by far the most produced Rosaceous fruit with over 93 million tonnes (Mt), followed by pear (~ 25 Mt), peach (~ 25 Mt), strawberry (> 9 Mt), almond (~ 4 Mt) and raspberry with just under 1 Mt grown in 2021 (FAOSTAT, 2023)

The Rosaceae family is an extraordinarily diverse plant family comprised of over 3000 species that belong to over a 100 genera and contain many important fruit, nut and ornamental crops (Shulaev et al. 2008). The genera *Malus* (apple), *Pyrus* (pear), *Prunus* (peach, plum, apricot, cherry and almond), *Fragaria* (strawberry), *Rubus* (raspberry, blackberry) and *Rosa* (rose) comprise most of the important species of this family, some of which have been used as food by human populations for over 7000 years (Shulaev et al. 2008). They are of major importance for the modern human diet as an abundant source of dietary fibre, vitamins, minerals and phytonutrients. Phytonutrients present in Rosaceous crops, such as polyphenolic compounds, play an important role in maintaining health and preventing disease (Ogah et al. 2014; Serra et al. 2020), with studies showing beneficial effects on cardiovascular diseases, inflammation, neurodegenerative disorders, cancer, obesity and interactions with gut microbiota (Cory et al. 2018; Di Lorenzo et al. 2021). Economically, the Rosaceae is the third most important plant family in the temperate region (FAOSTAT, 2023), with the world production steadily increasing for the past 20 years to meet the intense demand (Fig. 1).

**Fig. 2:**
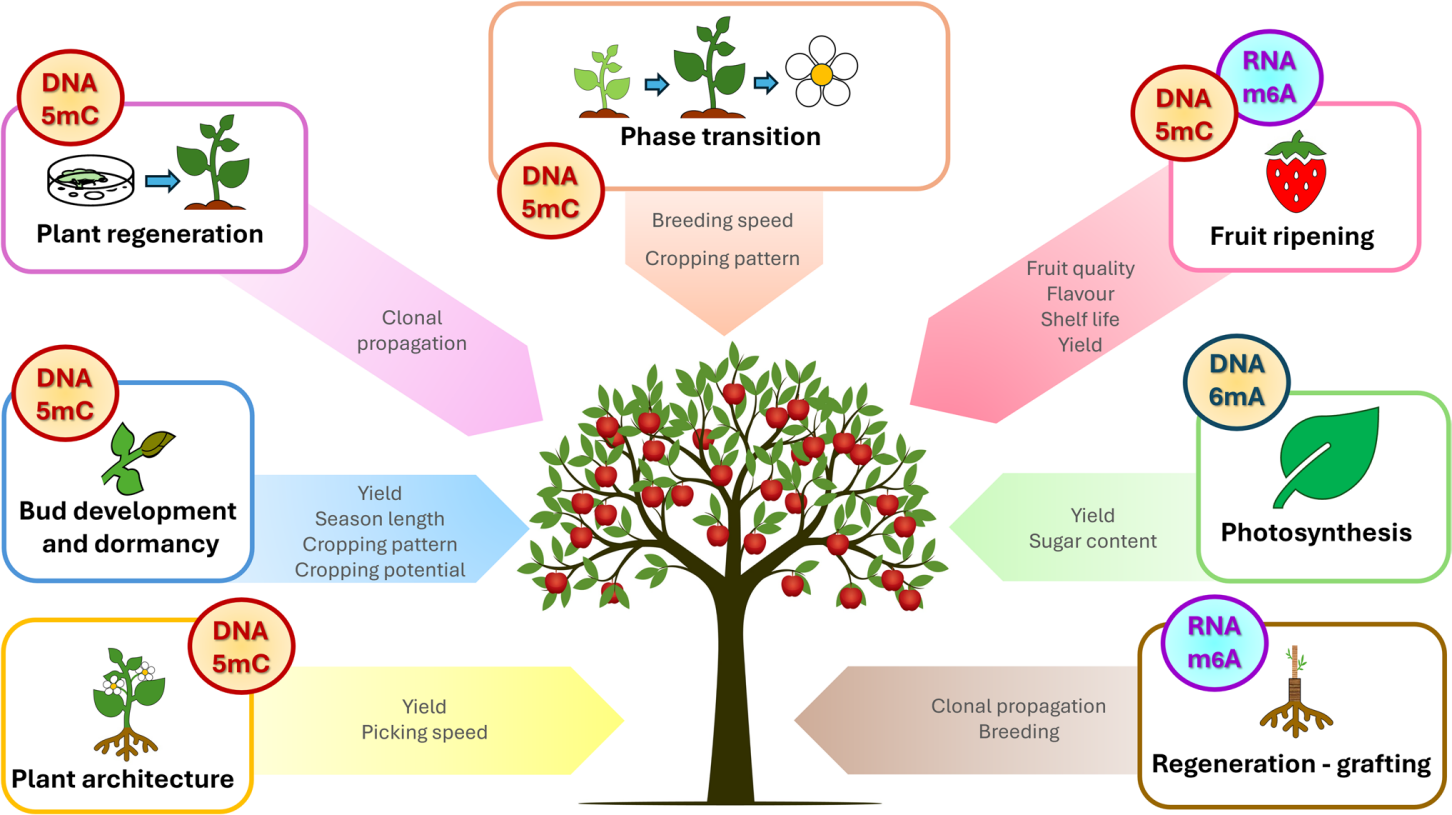
Developmental processes in Rosaceae affected by epigenetic methylation. DNA 5 mC methylation is involved in phase transition, plant regeneration, bud development and dormancy, fruit ripening and plant architecture; DNA N6-adenosine methylation has been associated with photosynthetic genes, whilst RNA m^6^A methylation of cellular transcripts plays a role in fruit ripening and grafting. These biological processes in turn affect traits and practices important for production of Rosaceous crops

Rosaceae are predominantly perennial plants many of which are trees or shrubs with complex flowering and cropping patterns that are influenced by endogenous and environmental cues. They are usually propagated clonally for agricultural production, most commonly through tissue culture, grafting, stolons or roots and have an extraordinary spectrum of distinct fleshy or dry fruit types derived from different parts of the flower (Liu et al. 2020). Understanding biological processes and the underlining molecular mechanisms that guide the diverse developmental traits and responses in the Rosaceae is paramount for continual production of these highly nutritious crops in a changing climate. Epigenetic methylation plays a role in many crucial developmental processes in Rosaceae that have direct relevance for their breeding and production (Fig. 2). Although this area of research is still relatively young, a growing interest has produced a plethora of studies and for several developmental processes, in-depth knowledge is now being accrued.

In the past 20 years molecular resources for Rosaceous crops have been steadily accumulating and efforts have been made to bridge the gap between DNA molecular studies and traditional crop breeding (Peace 2017; Iezzoni et al. 2020). New technologies and recent advances in molecular research have now opened further opportunities for facilitated crop breeding, such as increasing genetic gains through genomic selection, targeting specific traits with precision breeding by CRISPR-Cas9 gene editing or implementing epigenome modifications to create new, heritable phenotypes. Modifying the epigenome can be achieved through the use of chemicals, tissue culture, biotic and abiotic stresses, molecular RNA-based methods and through CRISPR-Cas editing by fusion of epigenetic effector domains to dead Cas (dCas) proteins (Pan et al. 2021; Dalakouras and Vlachostergios 2021). Utilising the epigenome as a source of variation to obtain novel plant phenotypes, to improve current varieties or to increase variation in crops with limited genetic diversity has become a promising strategy that could assist breeding to improve performance and yield of crops, as well as to potentially aid in adaptation to the globally changing environment (Dalakouras and Vlachostergios 2021; Varotto et al. 2020). Epigenetic studies in Rosaceae are necessary for unravelling the biology of agriculturally important traits and a number of investigations have recently begun to focus on epigenetic mechanisms in Rosaceous species and their involvement in the Rosaceae development.

DNA modification via methylation of the cytosine C-5 position (5mC), commonly referred to as “DNA methylation”, is involved in a wide range of plant biological functions including gene regulation, transposon suppression, imprinting and genome stability, and is relevant for a plethora of plant physiological and developmental processes (Mette et al. 2000; Shibuya et al. 2009; Nuthikattu et al. 2013; Satayaki and Gehring 2017; Pappalardo and Barra 2021). However, with recent advances in molecular technologies, additional chemical modifications found on DNA and RNA have been uncovered as new layers of epigenetic regulation (Arribas-Hernández and Brodersen 2020; Liang et al. 2020). In this article, we first summarise plant 5mC DNA methylation mechanisms and their components identified in Rosaceae species, and then review and discuss the role of 5mC DNA methylation in Rosaceae developmental processes, such as phase transition, bud development and dormancy, plant architecture, plant regeneration, fruit development, ripening and senescence. We then review recent advances in understanding *N*^*6*^-adenosine methylation of DNA (6mA) and RNA (m^6^A) as newly discovered additional layers of epigenetic control and discuss their emerging roles in plant development including recent findings in Rosaceous species.

### DNA 5mC methylation mechanisms in plants and their components in the Rosaceae

Cellular DNA methylation is a product of dynamic mechanisms that involve establishment, maintenance and removal of DNA methylation marks. Unlike the process of maintenance of DNA methylation, the establishment of plant 5mC DNA methylation involves cross-talk with small interfering RNA (siRNA) pathways and represents a sequence-specific methylation mechanism. It is achieved through the process of RNA-directed DNA methylation (RdDM) that relies on two plant-specific RNA polymerases (Pol): Pol IV and Pol V, which are evolutionarily derived from Pol II (Matzke and Mosher 2014; Zhang et al. 2018a). Plant DNA methylation processes have been thoroughly studied in *Arabidopsis thaliana,* and therefore, the summary of mechanisms and regulatory pathways in this review relies predominantly on the findings in this species.

**Fig. 3:**
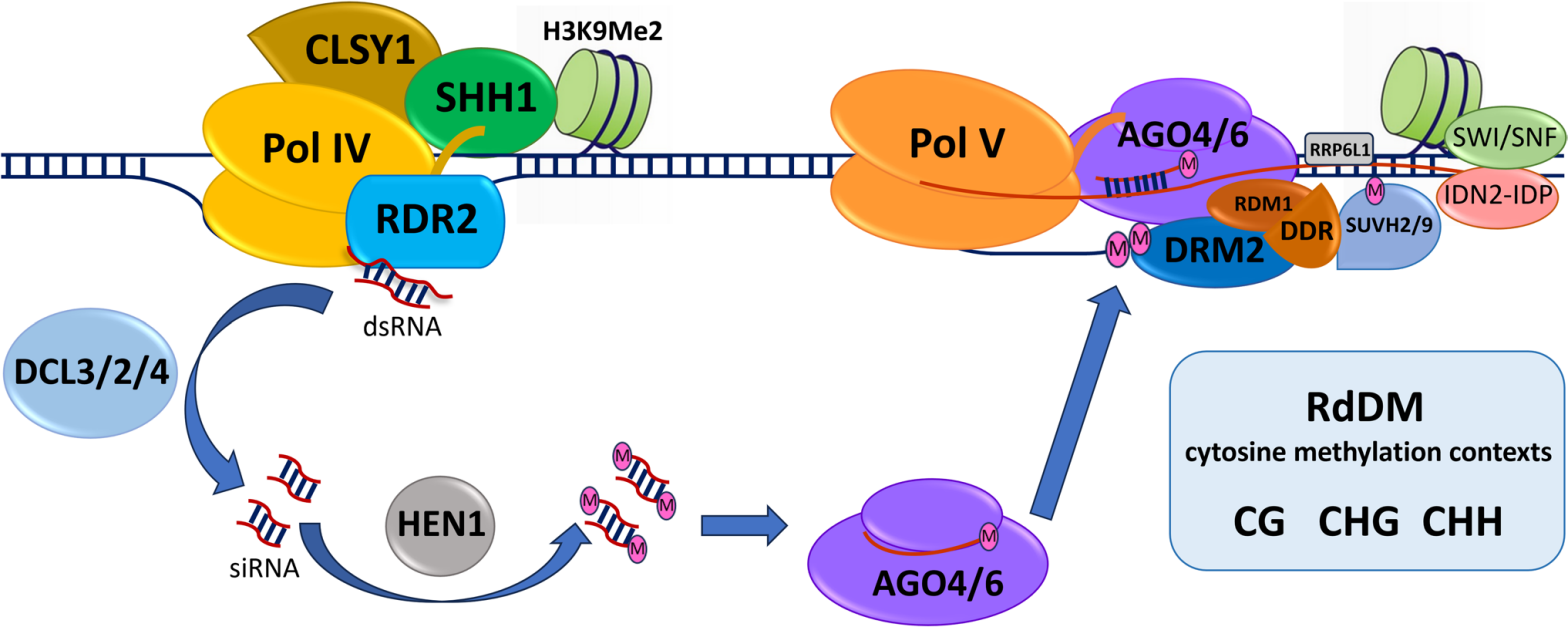
Establishment of plant DNA methylation in *A. thaliana* through the canonical RdDM. RNA polymerase IV (Pol IV) is recruited to specific sites on the genome based on pre-existing chromatin status, including H3K9Me2 mark recognised by SHH1 with which Pol IV interacts. CLSY1 is a chromatin remodeller also required for the process. Pol IV produces a short single-stranded RNA transcript which RDR2 turns into short dsRNA molecules. DCL 2,3 and 4 cleave the dsRNA to produce siRNA that are then stabilised by 3’ methylation by HEN1 and loaded into AGO effectors, AGO4 or AGO6. The loaded AGO complex is then able to interact in a sequence-specific manner with the nascent transcript of Pol V (represented by a red line) at a subset of genomic loci where Pol V is recruited to. AGO4 also interacts directly with the carboxy-terminal domain of Pol V and with DRM2 methyltransferase to initiate sequence-specific DNA methylation on the corresponding DNA strand. Pol V recruitment to genomic sites requires the chromatin remodeller complex DDR which interacts with SUVH2 and SUVH9 that recognise pre-existing DNA methylation. SWI/SNF chromatin remodelling complex adjusts nucleosome positioning and interacts with IDN2 and IDP complex that stabilises the RNA scaffold

*Establishment of 5mC DNA methylation.* In the canonical RdDM, siRNA biogenesis initiates with Pol IV recruitment to a specific region which is facilitated by pre-existing chromatin modifications (Fig. 3).

This includes interaction with SAWADEE HOMEODOMAIN HOMOLOGUE 1 (SHH1) which binds to dimethylated histone H3 lysine 9 (H3K9me2) and also to chromatin remodelling protein SNF2 DOMAIN-CONTAINING PROTEIN CLASSY 1 (CLSY1) (Zhang et al. 2013; Law et al. 2013). Pol IV transcription yields short non-coding RNAs of up to 50 nucleotides that are copied by RNA-DEPENDENT RNA POLYMERASE 2 (RDR2) into short double-stranded RNAs (dsRNAs) which are then cleaved by DICER-LIKE endoribonucleases (DCL) to generate predominantly 24nt siRNA molecules. The resulting siRNAs are stabilised by 3’ end methylation by HUA ENHANCER 1 (HEN1) (Ji and Chen 2012) prior to being incorporated into ARGONAUTE (AGO) effectors, AGO4 or AGO6. The AGO effectors then direct DNA methylation of siRNA targeted loci by sequence-specific pairing with the nascent RNA transcript produced by Pol V. The pairing is assisted by interaction between AGO4 and the carboxy-terminal domain of the largest subunit of Pol V called nuclear RNA polymerase E (NRPE) that contains the AGO “hook”. Within this complex, AGO4 interacts with DOMAINS REARRANGED METHYLASE 2 (DRM2) methyltransferase that then methylates the corresponding DNA in a sequence-specific manner (Zhong et al. 2014). This interaction is facilitated by the RNA-DIRECTED DNA METHYLATION 1 (RDM1) protein (a component of the chromatin remodelling complex known as DDR) that also associates with single-stranded methylated DNA (Gao et al. 2010). Additional protein factors are needed to stabilise the complex or facilitate recruitment of Pol V and interaction with chromatin marks. Appropriate occupancy of Pol V across the genome is facilitated by SUPPRESSOR OF VARIEGATION 3–9 HOMOLOG PROTEIN 2 (SUVH2) and SUVH9 that are able to recognise previously methylated DNA and assist in recruiting Pol V (Johnson et al. 2017). These two proteins also interact with the chromatin remodelling DDR complex (Gao et al. 2010). Further to this, SWITCH/SUCROSE NONFERMENTING (SWI/SNF) chromatin remodelling complex is also needed for RdDM: it alters nucleosome positioning and interacts with INVOLVED IN DE NOVO 2 (IDN2) and INVOLVED IN DE NOVO PARALOGUE (IDP) complex that stabilises the RNA scaffold. Protein RRP6-LIKE 1 (RRP6L1) helps retain Pol V non-coding transcript on the chromatin and also stabilises the Pol V scaffold (Zhang et al. 2014).

In addition to the canonical pathway described above that relies on Pol IV and RDR2 for siRNA biogenesis, RdDM can function through other molecular routes. Most notably, there is a non-canonical RdDM that predominantly targets transcribed transposable elements (TE) (Nuthikattu et al. 2013; McCue et al. 2015). It involves transcription by Pol II that can provide scaffold RNAs, and association with RNA-DEPENDENT RNA POLYMERASE 6 (RDR6) to produce precursors of 21-nucleotide or 22-nucleotide siRNAs. These siRNA have been shown to associate with AGO6 and mediate DNA methylation of the activated transposon loci (Nuthikattu et al. 2013; McCue et al. 2015).

Many of the major components of RdDM have been investigated in the Rosaceae. In the genus *Fragaria*, published sequences are available for Pol IV NRPD1 subunit, Pol V NRPE1 subunit, RDR2, RDR6, AGO4, AGO6, DCL3 and DRM2. Additionally, studies on the *Malus*, *Pyrus* and *Prunus* genera have also reported on many of the components of the RdDM (Table 1). Additional protein factors that make up the Pol V complex, RDM1, and a recently identified RdDM factor postulated to interact with IDN2: FACTOR OF DNA METHYLATION 1 (FDM1), have also been described in Rosaceae. However, most of the Rosaceae proteins involved in the interplay between RdDM and existing chromatin modifications are yet to be reported in the literature.

**Table 1:** Summary of 5mC DNA methylation components identified in Rosaceous species

Process	Gene description	Gene symbol	Function	*Arabidopsis* gene	Rosaceae species where orthologs have been identified														Reference
					*Fragaria vesca*	*Fragaria* × *ananassa*	*Malus domestica*	*Malus* cv. *spp*	*Pyrus communis*	*Pyrus bretschneideri*	*Pyrus sp.*	*Prunus avium*	*Prunus armeniaca*	*Prunus dulcis*	*Prunus mume*	*Prunus persica*	*Rosa chinensis*	*Rubus occidentalis*	
RdDM	POL IV SUBUNIT 1	*NRPD1*	Generates short non-coding RNAs that serve as template to produce double-stranded RNAs	AT1G63020		+						+	+						(Cheng et al. 2018; Canton et al. 2021)
	POL V SUBUNIT 1	*NRPE1*	Generates long non-coding RNA transcripts that serve as scaffold to bind the AGO-siRNA complex	AT2G40030		+													(Cheng et al. 2018)
	RNA-dependent RNA polymerase 2	*RDR2*	Generate double-stranded RNAs from single-stranded RNA templates	AT4G11130	+	+	+					+		+		+	+		(Cheng et al. 2018; Bélanger et al. 2022; Jing et al. 2023)
	RNA-dependent RNA polymerase 6	*RDR6*		AT3G49500	+	+	+					+		+		+	+		(Cheng et al. 2018; Bélanger et al. 2022; Jing et al. 2023)
	Argonaute 4	*AGO4*	Bind to small non-coding RNAs leading to the formation of the RISC complex that enables transcriptional gene silencing	AT2G27040	+	+	+					+	+	+		+	+		(Mirzaei et al. 2014; Xu et al. 2016; Cheng et al. 2018; Canton et al. 2021; Bélanger et al. 2022; Jing et al. 2023)
	Argonaute 6	*AGO6*		AT2G32940	+	+	+					+		+		+	+		(Mirzaei et al. 2014; Xu et al. 2016; Cheng et al. 2018; Bélanger et al. 2022; Jing et al. 2023)
	Dicer-like 3	*DCL3*	Cleavage of double-stranded RNA into 24nt siRNAs	AT3G43920	+	+	+		+			+	+	+		+	+	+	(Cheng et al. 2018; Bélanger et al. 2022; Belal et al. 2022; Jing et al. 2023)
	Domains Rearranged Methylase 2	*DRM2*	Methylates de novo targeted DNA sequences through its interaction with AGO4 or AGO6	AT5G14620	+		+	+		+			+		+	+			(Gu et al. 2016; Rothkegel et al. 2021; Canton et al. 2021; Xing et al. 2021)
	RNA-Directed DNA Methylation 1	*RDM1*	Binds single-stranded methylated DNA and associates with AGO4 and DRM2	AT3G22680				+											(Xing et al. 2021)
	HUA ENHANCER 1	*HEN1*	Adds methyl group to the 3’ end of the siRNAs	AT4G20910	+		+					+		+		+	+		(Bélanger et al. 2022)
Maintenance of methylation	Methyltransferase 1	*MET1*	Maintains CG methylation	AT5G49160	+	+	+			+		+	+		+	+			(Giannino et al. 2003; Gu et al. 2016; Cheng et al. 2018; Rothkegel et al. 2021; Canton et al. 2021)
	Chromomethylase 3	*CMT3*	Maintains CHG methylation	AT1G69770	+	+	+			+			+		+	+			(Gu et al. 2016; Cheng et al. 2018; Wang et al. 2021; Rothkegel et al. 2021; Canton et al. 2021)
	Chromomethylase 2	*CMT2*	Maintains CHH methylation and CHG methylation to a lesser extent	AT4G19020		+	+						+			+			(Cheng et al. 2018; Zhu et al. 2020b; Wang et al. 2021; Canton et al. 2021)
	Domains Rearranged Methylase 2	*DRM2*	Maintains methylation in all cytosine contexts	AT5G14620	+		+	+		+			+		+	+			(Gu et al. 2016; Rothkegel et al. 2021; Canton et al. 2021; Xing et al. 2021)
	Factor of DNA Methylation1	*FDM1*	Postulated to form a complex with IDN2 and regulate DNA methylation levels at target loci	AT1G15910	+														(Zheng et al. 2023)
	Decreased DNA Methylation 1	*DDM1*	Nucleosome remodeller, allows other methyltransferase enzymes to access DNA and is crucial for maintaining methylation	AT5G66750			+					+	+			+			(Xu et al. 2018; Canton et al. 2021)
Demethylation	Repressor of Silencing 1	*ROS1*	Effectuate active DNA demethylation through the base excision repair pathway: erase methylated cytosines irrespective of the sequence context	AT2G36490	+		+								+	+	+		(Ma et al. 2018; Rothkegel et al. 2021; Yu et al. 2022; Zheng et al. 2023)
	Transcriptional Activator Demeter	*DME*		AT5G04560	+		+				+		+		+	+			(Gu et al. 2016; Liu et al. 2018; Wang et al. 2021; Rothkegel et al. 2021; Canton et al. 2021)
	Demeter-like Protein 2	*DML2*		AT3G10010	+		+			+					+	+			(Gu et al. 2016; Zhu et al. 2020b; Rothkegel et al. 2021)
	Demeter-like Protein 3	*DML3*		AT4G34060	+		+			+					+	+			(Gu et al. 2016; Zhu et al. 2020b)

**Fig. 4:**
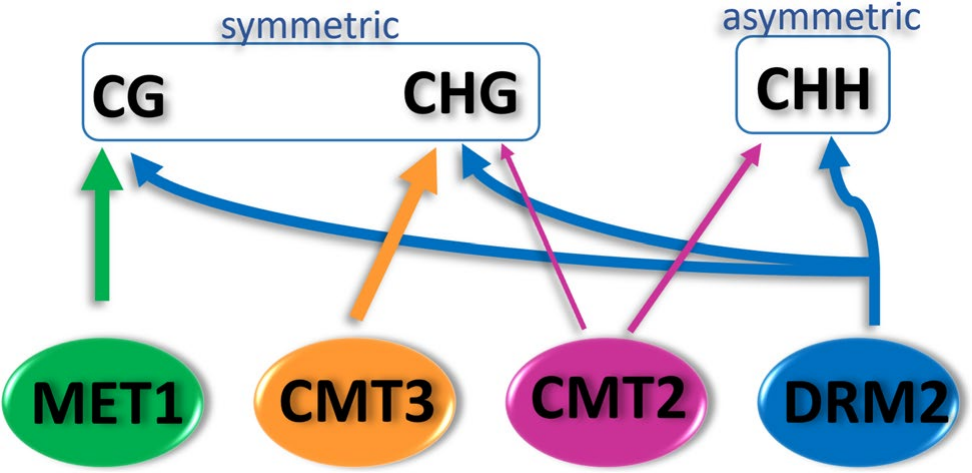
Enzymes involved in maintenance of DNA methylation. CG, CHG and CHH (H = A, T, or C) refer to cytosine nucleotide context where the presence of G provides a cytosine on the opposite strand which can be symmetrically methylated. MET1 maintains methylation in the CG context; CMT3 is the main enzyme that maintains methylation in the CHG context, whilst CMT2 can methylate in CHG and CHH context. DRM2 methylates in all three contexts through RdDM

*Maintenance of 5mC DNA methylation.* RdDM methylates DNA through DRM2 in all three cytosine contexts, CHH, CHG and CG (where H stands for A,T or C), and is the only methylation mechanism that can both methylate de novo and maintain previously methylated sites (He et al. 2021). However, cytosine methylation in the symmetric CG and CHG contexts (i.e. contexts that provide cytosine on the opposite strand) can be faithfully reproduced upon replication by robust methylation maintenance mechanisms that do not require a siRNA trigger (Fig. 4).

Cytosine methylation in the CG context is maintained by the METHYLTRANSFERASE 1 (MET1) enzyme that methylates the cytosine on the newly synthesised, unmethylated strand following replication (Kankel et al. 2003), in an interplay that also requires the presence of METHYL-BINDING PROTEINS (MBP) and HISTONE DEACETYLASES (HDAC) (Zemach and Grafi 2003; Dalakouras and Vlachostergios 2021). Maintenance methylation in the CHG methylation context is achieved predominantly by the enzyme CHROMOMETHYLASE 3 (CMT3) (Lindroth et al. 2001) that binds H3K9me2 and forms a part of a regulatory feedback loop that functions to reinforce both H3K9me2 and DNA methylation. Another chromomethylase, CMT2 that also binds H3K9me2, is able to methylate cytosines in CHG and CHH context (Stroud et al. 2014). It is important to note that the maintenance mechanisms and the RdDM do not function homogeneously across the genome – for example, modifications of pericentromeric heterochromatin rely predominantly on maintenance mechanisms employing MET1, CMT2, CMT3 and a chromatin remodeller DECREASED DNA METHYLATION 1 (DDM1), whilst the RdDM mechanism plays a greater role in the euchromatic regions of the genome (Zemach et al. 2013; Matzke and Mosher 2014). In Rosaceae, the four maintenance DNA methyltransferases have been identified in *Malus*, *Fragaria* and *Prunus,* whilst in pear only sequences of MET1, CMT3 and DRM2 have been reported. The DDM1 chromatin remodeller has been reported in *Malus* and *Prunus* species (Table 1).

**Fig. 5:**
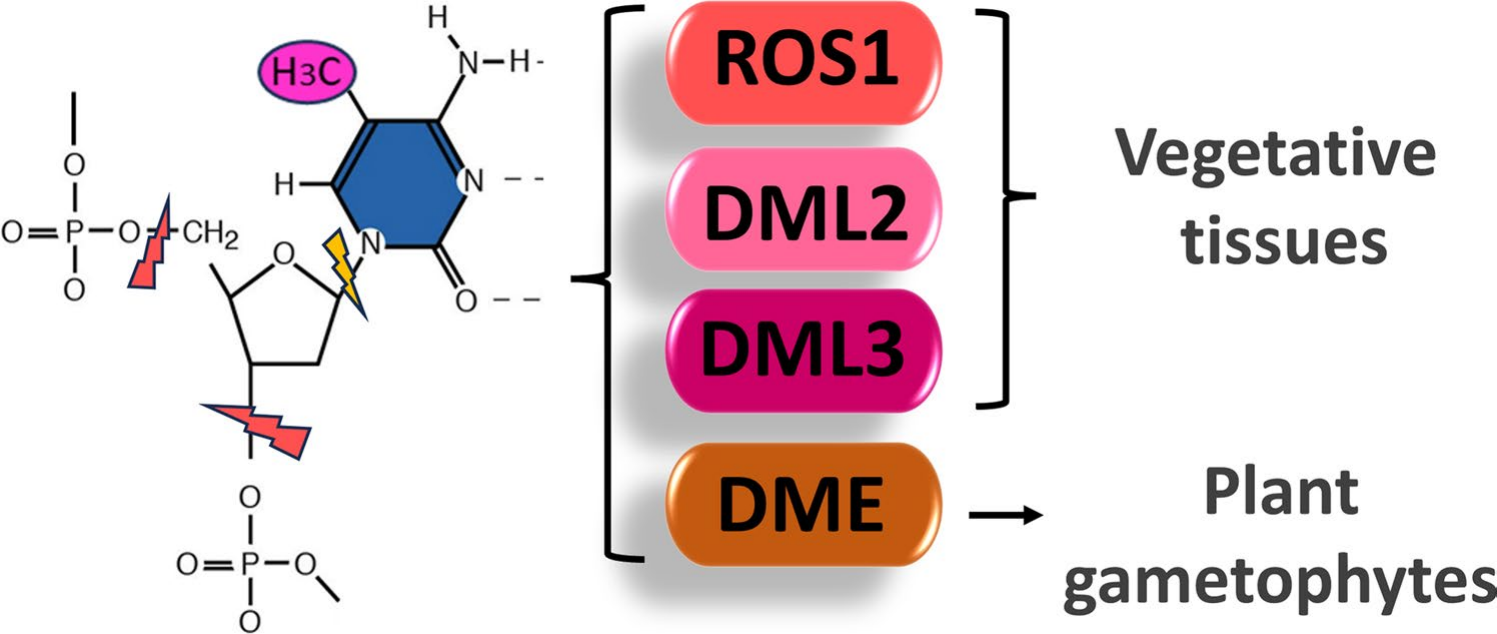
Enzymes and reactions involved in plant DNA demethylation. Plant DNA demethylases: ROS1, DME, DML2 and DML3, are bifunctional 5mC DNA glycosylases-apurinic/apyrimidinic lyases. They initially catalyse the removal of the methylated cytosine base (yellow bolt) which is followed by severing of the phosphate DNA backbone (red bolts) to create a gap which is then filled in by DNA polymerase and DNA ligase enzymes. ROS1, DML2 and DML3 are active in all vegetative tissues whilst DME has established roles in the gametophytes

*Active 5mC DNA demethylation.* In addition to the processes of establishment and maintenance, DNA methylation can also be passively or actively removed. Failure to maintain methylation at a site can lead to passive demethylation, whilst active DNA demethylation is achieved through a base excision repair pathway (Fig. 5).

In plants, DNA demethylase enzymes are a family of bifunctional 5mC DNA glycosylases, apurinic/apyrimidinic lyases. They initially remove the 5mC base, and in the next step eliminate the phosphate backbone bonds to produce an abasic site, and the gap which is formed is then filled by DNA polymerase and ligase enzymes (Gong et al. 2002; Lee et al. 2014). There are four DNA demethylase genes in *Arabidopsis*: *REPRESSOR OF SILENCING 1* (*ROS1*), *TRANSCRIPTIONAL ACTIVATOR DEMETER (DME), DEMETER-LIKE PROTEIN2* (*DML2*) and *DML3* (Gong et al. 2002; Gehring et al. 2006; Ortega‑Galisteo et al. 2008). ROS1, DML2 and DML3 function in all vegetative tissues, whilst DME, although found to be active vegetative tissues (Zeng et al. 2021), has a predominant importance in plant gametophytes. It is preferentially expressed in the central cell (female gametophyte) and in the vegetative cell (male gametophyte) and is involved in gene imprinting (Gehring et al. 2006; Penterman et al. 2007). Rosaceae demethylation enzymes have been investigated in *Fragaria*, *Malus* and *Prunus* where orthologues of all four *Arabidopsis* genes have been identified. Some of the genes have also been uncovered in *Pyrus* and *Rosa* (Table 1).

The presence of 5mC DNA methylation is most frequently considered a repressive transcriptional signal causing silencing in majority of cases, specifically when the promoter region of a gene has been targeted. However in some instances, promoter methylation has also been shown to activate transcription (Shibuya et al. 2009; Lei et al. 2015; Lang et al. 2017). It has been shown in *Arabidopsis* that transcriptional activation can be attributed to methyl reader proteins such as SUVH that specifically associate with DNA methylation and recruit DNAJ domain-containing proteins to form protein complexes that enhance proximal gene expression of methylated genes (Harris et al. 2018). One of the most notable cases where DNA promoter methylation activates gene expression is the transcriptional control of *ROS1* DNA demethylase. *ROS1* gene expression is downregulated in mutants of the RdDM pathway suggesting coordination between the mechanisms of DNA methylation and demethylation. The *ROS1* promoter region contains a 39 nucleotide sequence called the methylation monitoring sequence (MEMS) whose hypomethylation downregulates and hypermethylation activates *ROS1* transcription, thereby serving as a “methylstat” that senses and regulates genomic DNA methylation levels (Lei et al. 2015). Although the MEMS sequence itself and its methyl status have not been published on in Rosaceous species, a study in *Fragaria* (Zheng et al. 2023) has demonstrated that the general promoter region of the *ROS1* gene is hypomethylated and the gene is downregulated in an RdDM pathway mutant of *FDM1* (*fvefdm1*) – a finding which is consistent with the “methylstat” concept described in *Arabidopsis.*

### The involvement of 5mC DNA methylation in Rosaceae developmental events

DNA 5mC methylation is a fundamental epigenetic signature that is known to affect gene expression and therefore plays an integral part in our understanding of the molecular basis of growth and development in the Rosaceae family.

*Phase transition.* The life cycle of plants involves a transition between various developmental phases, from juvenile to adult, vegetative to reproductive and progression into senescence (Bäurle and Dean 2006). Before they become competent to flower and reproduce, plants go through a phase of vegetative growth when they tend to increase their photosynthetic capacity and their overall size. This vegetative growth is divided into a juvenile and an adult vegetative phase and during the adult vegetative phase plants acquire a capability to form reproductive organs and gain reproductive competence. Finally, the adult vegetative phase transitions to reproductive growth when the competent shoot apical meristem (SAM) responds to signals that trigger floral initiation (Huijser and Schmid 2011). Studies on model plants (*Arabidopsis* and tomato) have provided evidence for DNA methylation as an important regulatory mechanism underlying phase transitions (Burn et al. 1993; Finnegan et al. 1998; Yang et al. 2019). In Rosaceae, one of the first studies to relate DNA methylation to juvenile-to-adult transition was done in peach where it was found that juvenile (from seedlings) and juvenile-like (from basal shoots) SAMs exhibited lower nuclear DNA methylation levels compared to methylation of adult, competent SAMs (Bitonti et al. 2002), but more nuanced effects of methylation have been published since. A recent study on spatiotemporal methylation in apple (*Malus hupehensis)* revealed that, in addition to the well-known post-transcriptional regulation of juvenile-to-adult transition via miR156/miR172 targeting of *SPL* and *AP2* transcription factors (TFs), the mechanism employing these two TF gene families may also be affected by DNA methylation (Xing et al. 2020). The study reported that, whilst global DNA methylation levels were slightly higher in apple juvenile leaves than those of adult leaves in all three cytosine contexts, the identified differentially methylated regions (DMRs) showed that methylation level of seven *AP2* family genes was significantly higher in adult than in juvenile leaves and congruent with their downregulated expression. They also reported on other relevant TFs and found that methylation levels of *WRKY*, *NAC*, *ERF*, *WOX*, *KNAT*, *EIN3*, *SCL*, *ZAT*, and *HSF* genes were significantly higher in the adult leaves than in the juvenile ones, whilst *TCP*, *MADS-box* and *DOF* genes showed the opposite trend (Xing et al. 2020). Another study in apple showed that changes in 24nt siRNA were correlated with vegetative-to-floral transition. Namely, siRNA abundance was studied from vegetative and floral buds during flower induction in apple trees where more than 90% of the differentially expressed 24nt siRNAs showed stronger expression in a whole genome pattern in the floral buds, suggesting an increased level of RdDM DNA methylation during floral transition (Guo et al. 2017).

*Bud development.* DNA methylation may play a role in controlling apple floral bud development. Using proteomic profiling, Kofler et al. (2022) sought to understand the complex processes associated with the formation of floral buds in biennial apple by manipulating the cropping of two cultivars to mimic the “On” state (high crop load and inhibited floral bud formation) and the “Off” state (low crop load and a promoted formation of floral buds). They found that in the “Off” state the buds showed an increased abundance of histone and DNA methylation proteins suggesting the involvement of these epigenetic mechanisms in the flower bud induction process. In another study, global methylation and transcriptional analyses of apple buds with diverse flowering abilities (axillary buds with no flowering; long-shoot buds with low rate of flowering; and spur buds with a high flowering rate) reported a negative correlation between gene body methylation and transcript abundance of highly expressed flowering-associated genes in spur buds, and observed that genes with methylated promoters were generally more highly expressed than genes with unmethylated promoters, implying that promoter methylation, as an activating mark, may be involved in apple bud formation (Xing et al. 2019). In addition to this, studies of non-infectious bud failure attempted to relate DNA methylation in buds of different almond clones to this economically important condition for which no causal mechanism has yet been identified, and suggested that bud failure in almond might be associated with DNA hypomethylation at a number of genomic loci (Fresnedo-Ramírez et al. 2017; D’Amico-Willman et al. 2021).

*Bud dormancy.* Dormancy is a coping mechanism employed by plants to halt growth and development for a certain time period to enable survival in unfavourable conditions (Lang et al. 1987). Bud dormancy and its release depend on internal and environmental signals such as cold accumulation and photoperiod. Rosaceous species require sufficient winter chilling to release bud dormancy as well as warm temperatures to resume growth, and several lines of evidence have implicated DNA methylation as a regulatory mechanism in bud dormancy control in Rosaceae. In cherry and apple dormant buds, global methylation changes during chilling accumulation were associated with genes involved in cold sensing and signalling, oxidation–reduction processes, metabolic pathways as well as cell wall remodelling (Kumar et al. 2016; Rothkegel et al. 2020; Narváez et al. 2022). In addition to global methylation patterns, many studies on bud dormancy focused specifically on the Evergrowing (*EVG)* locus containing *DORMANCY-ASSOCIATED MADS-box* (*DAM*) genes that had been previously identified in *Prunus* species. In sweet cherry, bisulphite sequencing and methylated DNA immunoprecipitation analyses showed that the reduced expression of *DAM* genes coincided with DNA hypermethylation of the promoter region. When the chilling requirement was complete there was a marked increase in *DAM* gene *PavMADS1* promoter methylation, accompanied with an increase in matching siRNA abundance (Rothkegel et al. 2017). A comprehensive study in peach analysed how different temperature regimes affect and regulate the six linked *DAM* genes at the *EVG* locus (Zhu et al. 2020a). The study identified the role of multiple epigenetic events including histone H3 lysine 27 trimethylation (H3K27Me3), non-coding RNA (ncRNA) regulation and DNA methylation, and showed that during chilling, the six *DAM* genes were hypermethylated, and associated with the production of 24nt sRNA. Interestingly, CG methylation remained relatively constant during the chilling period but declined after shifting to warm temperature, whilst the warm temperature increased CHH methylation overall except for *DAM1* and was particularly prominent in *DAM4* and *DAM5*. Therefore, the data suggest that warm temperature regulates CG and CHH methylation in an opposing manner and indicate that warming may activate RdDM in the *DAM* region to enable flower development free from any residual DAM inhibition (Zhu et al. 2020a).

In addition to its involvement in bud dormancy release, DNA methylation in Rosaceae has also been associated with dormancy induction and endogenous signals such as phytohormones. A study in strawberry showed that the induction of strawberry dormancy is accompanied by a simultaneous increase in global DNA methylation and abscisic acid (ABA) levels, a decrease in indole-3-acetic acid (IAA) content and changes in transcript levels for related genes including *PIN-FORMED (PIN*) auxin transporter, *9-CIS-EPOXYCAROTENOID DIOXIGENASE* (*NCED*), *DRM* and *ROS1* (Zhang et al. 2012).

*Plant architecture.* DNA methylation has been shown to have an impact on plant architecture in Rosaceae. CRISPR-Cas9-mediated strawberry mutant lines of the *FDM1* component of the RdDM pathway displayed reduced DNA methylation and severe morphological abnormalities including small leaves, flowers and fruits (Zheng et al. 2023). The study also reported a decline in the phytohormone content (auxin and gibberellic acid) of these mutant lines compared to the wild type. In addition to this, in *Taihangia rupestris*, an endangered andromonoecious species of Rosaceae, DNA methylation was found to be involved in the formation of different types of flowers. Cytosine methylation was higher in the perfect flowers than in the staminate flowers and an increase of cytosine methylation levels was found to correlate with developmental stages in both flower types (Li et al. 2021).

*Plant regeneration.* Although plants undergo transitions from the juvenile to the adult phase, regeneration – a process which involves regaining some or all of the juvenile features, can also take place under certain conditions (Hackett and Murray 1993). Plant regeneration is a selective trait because it is essential for survival upon wounding (Lardon and Geelen 2020) and recent evidence has implicated DNA methylation in modulating plant regeneration in Rosaceae. Common methods employed for obtaining clones in Rosaceae crops include in vitro tissue culture and grafting (Aldwinckle and Malnoy 2009; Zhang et al. 2020). In strawberry tissue culture, the methylomes of leaf and callus tissues were compared and a DNA methylation increase was reported upon the formation of the callus from leaf tissue, predominantly in the CG and CHG context (Liu et al. 2022). The study also found that four methyltransferase genes and two *AGO* genes exhibited up-regulation in the callus relative to the leaf tissue thus providing evidence for potential involvement of DNA methylation in callus formation in *Fragaria vesca*. A study on a different species of wild strawberry, *Fragaria nilgerrensis,* that analysed successive tissue culture stages showed that the overall methylation levels alternately decreased and increased during the entire tissue culture process. CHH context accounted for the lowest proportion of total cytosine methylation but showed the most prominent methylation change, with most of the changes located in the transposable element regions (Cao et al. 2021). Interestingly, whilst the application of DNA methylation inhibitor, 5’-azacytidine (5-AzaC) on leaf explants inhibited callus formation and shoot regeneration in strawberry (Liu et al. 2022), in peach, it significantly increased callus development (Zheng et al. 2022). These findings suggest that different Rosaceous fruit species may employ diverse epigenetic responses during the tissue culturing process.

*Fruit ripening and senescence.* Fruit ripening involves thousands of genes that control various aspects of processes such as softening or lignification of specific cell layers, accumulation of sugars, acids, pigments and release of volatile compounds amongst others (Osorio et al. 2013). Recently, a series of findings have provided insights into the link between DNA methylation state and the expression of ripening-associated genes. In a study on strawberry ripening, a decrease in DNA methylation, a down-regulation of the RdDM-pathway-genes and an up-regulation of hormone-, anthocyanin- and flavour-related genes in *Fragaria* × *ananassa* was observed (Cheng et al. 2018). Moreover, treatment with DNA methylation inhibitor 5-AzaC induced early ripening in strawberry, demonstrating the impact of DNA demethylation. The study indicated that strawberry, as a non-climacteric fruit, similarly to tomato as a climacteric fruit, employs DNA hypomethylation during the ripening process; however, whilst tomatoes undergo global DNA demethylation (Lang et al. 2017), the hypomethylation observed in strawberry is related to RdDM downregulation (Cheng et al. 2018).

Development of colour and flavour are key processes during fruit ripening. Peel colour is an important visual attribute of fruit and differences in the promoter methylation level of *MYB10*, a regulator of the anthocyanin pathway, have been shown to affect coloration pattern and the formation of green- and red-skinned sports in pear (Wang et al. 2013; Qian et al. 2014) and yellow-skinned sports in apple (Telias et al. 2011; Jiang et al. 2020), respectively. The release of flavour compounds during ripening is of utmost importance for Rosaceous fruit quality and an important target for breeding. A recent study analysed the role of DNA methylation in affecting furanone biosynthesis in strawberry. Furanone imparts a caramel-like and fruity-floral aroma and is an essential component of strawberry flavour. The study found that the DNA methylation level of the strawberry QUINONE OXIDOREDUCTASE 3 (*FaQR3*) promoter was negatively correlated with *FaQR3* expression and furanone accumulation. Downregulation of *FvAGO4* led to upregulation of *FaQR3* expression and increased furanone content implicating the RdDM pathway in accumulation of flavour compounds in strawberry (Li et al. 2023). In peach, linalool is one of the main contributors of its characteristic aroma, and its synthesis is associated with terpene synthase (TPS) enzymes (Eduardo et al. 2010). A recent study that used bisulfite sequencing, found that DNA hypomethylation of *TPS3* gene led to an increase in transcript level and an accumulation of linalool during peach ripening (Wei et al. 2021).

The process of senescence is a major transition event that leads to the deterioration and degeneration of plant parts or the whole plant (Pérez-Llorca and Munné-Bosch 2021) and marks the final stage the fruit ripening process. Harvested strawberries displayed a delayed senescence in response to 5-AzaC (Chen et al. 2023) and the induced hypomethylation led to changes in gene expression with an impact on strawberry postharvest quality such as reduced soluble solid accumulation, delayed colouration, low softening rate and longer shelf life (Chen et al. 2023). In another study on strawberry, transcriptome analyses showed that key genes responsible for the biosynthesis of anthocyanins, phenylpropanoids and hormones such as abscisic acid (ABA) were affected by 5-AzaC blocking of methylation, and that the treatment brought about downregulation of genes associated with ABA biosynthetic genes, but upregulated genes associated with its degradation (Martínez-Rivas et al. 2022).

*Other processes.* In addition to the involvement in ripening and senescence, the roles of DNA methylation have been demonstrated in two other fruit-associated qualities important for consumer acceptance: flesh browning and mealiness. An increase in DNA methylation and an up-regulation of *CMT* genes was observed in a study on apple browning as the flesh browning progressed, and in the same study, the use of the DNA methylation inhibitor 5-AzaC resulted in a delay in observed browning in apple (Wang et al. 2021). DNA methylation changes have also been shown to have an impact on fruit mealiness in peach with mealy fruits exhibiting higher methylation levels than control fruits, particularly in the CHH context (Rothkegel et al. 2021).

The studies reviewed above show that 5mC DNA methylation is implicated in a plethora of Rosaceous developmental processes. Whilst many of the initial studies have been correlative in nature, the current and future research efforts are likely to investigate DNA methylation dynamics in Rosaceae in more mechanistic detail.

### DNA N^6^-adenosine methylation (6mA) and its emerging roles

DNA *N*^6^-adenosine methylation (6mA) is another type of modification that has recently been shown to be a regulatory DNA epigenetic mark found in plants (Liang et al. 2018a, 2020). In contrast to 5-methylcytosine, 6mA modification is less well understood in eukaryotes. Whilst in human studies, the components of the 6 mA machinery have been identified and characterised, these mechanisms in plant models remain largely obscure. Nevertheless, with the advent of highly sensitive sequencing platforms 6mA genomic distribution has been reported in plants, including *Arabidopsis* (Liang et al. 2018b), *Fragaria vesca* (Xie et al. 2019; Liu et al. 2019), *Oryza sativa* (Zhou et al. 2018; Zhang et al. 2018b), *Hippophae rhamnoides* (Zhang et al. 2022) and *Rosa chinensis* (Liu et al. 2019), and this methylation mark has thus far been implicated in photosynthesis, flowering and stress response (Jiménez-Ramírez et al. 2022). Therefore, despite the paucity of studies on 6mA, some information is available for Rosaceous plants. In *F. vesca*, like in *Arabidopsis* (Liang et al. 2018b) the 6mA distribution was found to be enriched in the coding regions and positively correlated with gene expression. Furthermore, high 6mA density loci were found to be associated with photosynthesis, plastid and thylakoid development-related genes (Xie et al. 2019; Liu et al. 2019) and 6mA density was shown to be significantly higher in long non-coding RNA (lncRNA) than in protein-coding genes (Xie et al 2019). Further studies are needed to fully understand the full relevance of this DNA modification in plant physiological and developmental processes and to unravel the mechanisms of 6mA DNA methylation in plants.

### Epitranscriptome: RNA N^6^-adenosine methylation (m^6^A) mechanism and its components in Rosaceae

Covalent nucleotide modifications are a critical way of controlling the processing, localisation, stability, and translatability of mRNAs. Recently, RNA *N*^6^-methylation of adenosine (m^6^A) and other modifications, such as 5-methylcytidine (m^5^C), *N*^1^-methyladenosine (m^1^A) and pseudouridine (Ψ), have been uncovered as novel epigenetic regulators following advances in high-throughput detection methods (Nie et al. 2022; Lang et al. 2023). Of all the newly identified chemical modifications, m^6^A is the most widespread and best elucidated RNA modification to date (Wiener and Schwartz 2021; Zaccara et al. 2023) and makes up 80% of eukaryotic RNA methylation (Kierzek and Kierzek 2003). This modification is present in coding and non-coding RNA (Nie et al. 2022; Lang et al. 2023) and has been implicated in mRNA stability (Guo et al. 2022) and transposon suppression (Fan et al. 2022).

Despite having been initially described in mammalian systems, the m^6^A machinery, which consists of writers, erasers and readers, has been found to be conserved in eukaryotic kingdoms (Zhong et al. 2008; Růžička et al. 2017; Arribas-Hernández and Brodersen 2020). Like DNA methylation, m^6^A RNA methylation is reversible due to the activities of the writers and erasers. The writers (methyltransferase enzymes) have been shown to catalyse the installation of the m^6^A mark to the consensus RNA sequence RRACH (where R = A/G; H = U/A/C) (Schibler et al. 1977), and more recently, a conserved UGUAH (H = A/U/C) sequence has been identified in *Malus* (Mao et al. 2021; Xu et al. 2023a) which is comparable with the URUAY (R = A/G; Y = U/C) motif detected in *Arabidopsis* and tomato (Wei et al. 2018; Zhou et al. 2019). Another characteristic of m^6^A methylation is its conserved distribution around the stop codon and 3’ UTR region which has been uncovered in *Arabidopsis*, as well as in crops including strawberries, apple, tomato, rice and maize (Zhou et al. 2019, 2021; Miao et al. 2022; Xu et al. 2023a). m^6^A depositions have been observed in the coding regions; however, the m^6^A methylation here is less prominent than at the 3’ UTR (Zhou et al. 2019, 2021; Xu et al. 2023a).

**Fig. 6:**
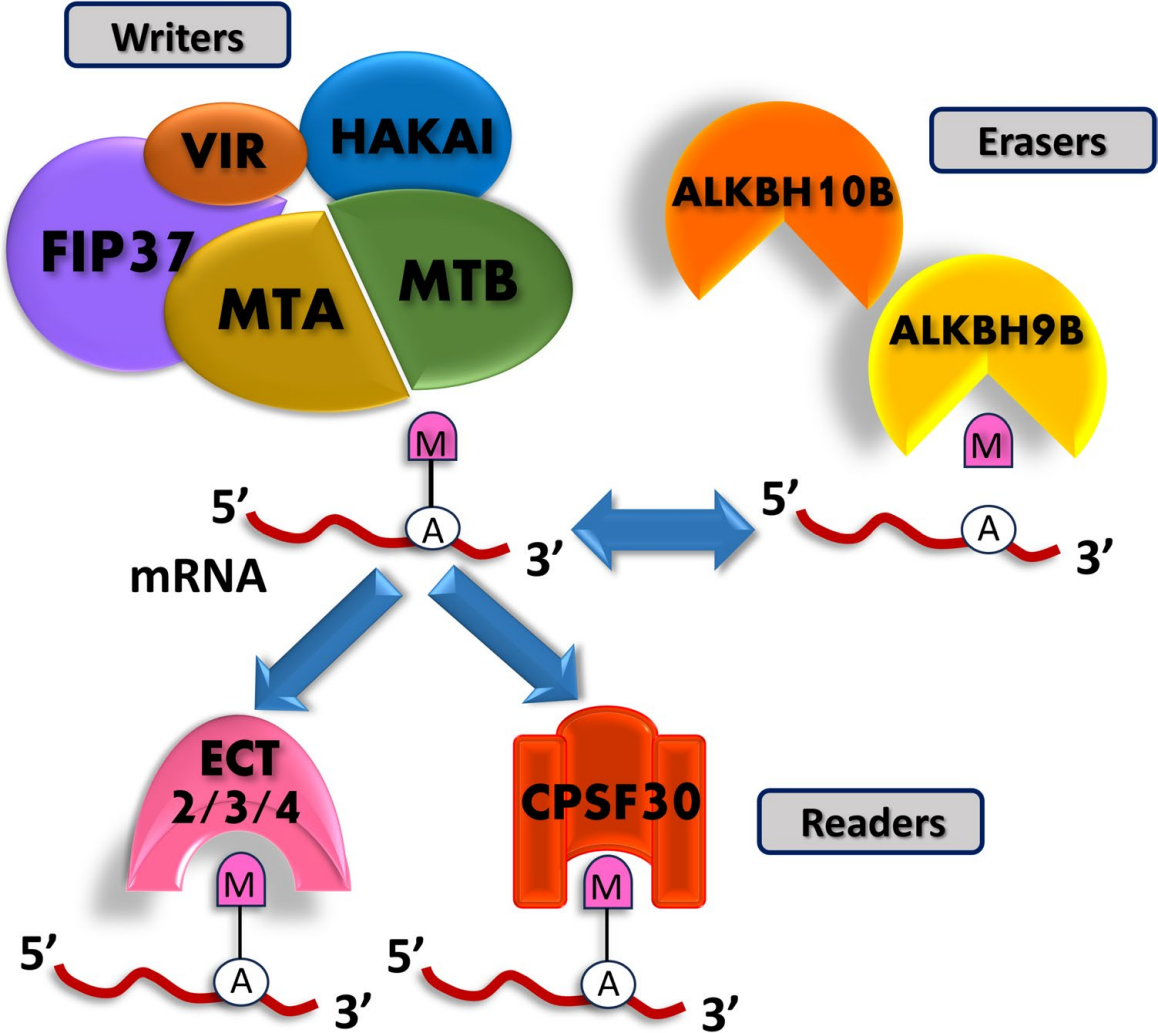
Components of the RNA *N*^6^-adenosine methylation mechanism in plants. Writers: methyltransferase-like proteins MTA and MTB, the splicing factor FIP37, VIR protein and a putative ubiquitin E3 ligase HAKAI are believed to form the writer complex. Erasers: ALKBH oxidases with demethylase activity ALKBH9B and ALKBH10B. Readers: YTH family proteins ECT 2, 3 and 4, and CPSF30

Although the biochemistry of the m^6^A writer reaction is simple, the major eukaryotic mRNA adenosine methyltransferase comprises a complex molecular machinery (Arribas-Hernández and Brodersen 2020). The main components in plants are methyltransferase-like proteins MTA and MTB (homologues to mammalian METTL3 and METTL14, respectively) that require several additional factors to fulfil their role (Fig. 6).

These include the splicing factor FKBP12 INTERACTING PROTEIN 37 (FIP37), the protein VIRILIZER (VIR) and the putative ubiquitin E3 ligase HAKAI. The m^6^A erasers, on the other hand, are a family of ALKBH oxidases that have demethylase activity and are associated with the removal of the epitranscriptomic mark, whilst the readers are YT521-B HOMOLOGY DOMAIN (YTH) proteins that recognise the adenosine *N*^6^-methyl group via a highly conserved hydrophobic binding pocket (Theler et al. 2014). In plants some of the YTH readers are known as *EVOLUTIONARILY CONSERVED C-TERMINAL REGION* (*ECT*) family genes (Arribas-Hernández and Brodersen 2020), whilst another identified YTH reader is the 30-kD subunit of the CLEAVAGE AND POLYADENYLATION SPECIFICITY FACTOR (CPSF30) (Pontier et al. 2019). Writers, erasers and readers with close homology to their mammalian counterparts have been reported in *Arabidopsis* (Arribas-Hernández and Brodersen 2020; Nishat et al. 2022), and the same groups are also present in Rosaceous species (Nie et al. 2022; Han et al. 2023; Lang et al. 2023). The components identified in Rosaceous species are listed in Table 2.

**Table 2:** Distribution of putative m^6^A effectors identified so far in Rosaceous species

Species	m^6^A									Reference
	Writers						Erasers	Readers		
*Homo sapiens*	*METTL3*	*METTL14*	*METTL4*	*WTAP*	*KIAA1429*	*HAKAI*	*ALKBH* and *FTO*	*YTH*		
A*rabidopsis thaliana*	*AtMTA* (1)	*AtMTB* (1)	*AtMTC* (1)	*AtFIP37* (1)	*AtVIRILIZER* (1)	*AtHAKAI* (1)	*ALKBH* (13)	*ECT* (12)	*CPS30* (5)	(Liang et al. 2020; Nishat et al. 2022; Han et al. 2023)
*Fragaria vesca*	1	1	1	1	1	1	9	8	1	(Xu et al. 2023b; Han et al. 2023; Lang et al. 2023)
*Malus domestica*	2	1	1	1	2	2	14	15	2	(Nie et al. 2022; Han et al. 2023; Lang et al. 2023)
*Prunus avium*	2	1	1	1	-	1	10	9	1	(Nie et al. 2022; Han et al. 2023)
*Prunus dulcis*	1	1	-	3	-	1	7	7	6	(Nie et al. 2022)
*Prunus mume*	1	1	1	3	1	1	10	7	1	(Han et al. 2023)
*Prunus persica*	1	1	1	2	1	1	11	7	1	(Nie et al. 2022; Han et al. 2023)
*Pyrus betulifolia*	2	2	1	2	2	2	14	13	2	(Han et al. 2023)
*Pyrus bretschneideri*	2	2	2	1	2	2	8	15	2	(Han et al. 2023)
*Pyrus communis*	1	2	1	1	1	2	10	14	2	(Han et al. 2023)
*Rosa chinensis*	2	1	-	1	-	1	5	9	8	(Nie et al. 2022)
*Rubus occidentalis*	1	1	1	1	1	1	10	8	1	(Han et al. 2023)

### RNA m^6^A in plant development and its roles in Rosaceae

Studies on *Arabidopsis* and tomato are starting to unravel the biological roles of m^6^A in plant development (Zhou et al. 2019; Arribas-Hernández and Brodersen 2020). In tomato m^6^A mRNA methylation, which shows dynamic changes during fruit ripening, is generally negatively correlated with transcript abundance and exhibits molecular cross-talk with DNA 5 mC methylation (Zhou et al. 2019). A recent study in apple has shown that this association between m^6^A and 5 mC methylation may also be a feature of Rosaceae (Xu et al. 2023a) and findings in strawberry show association between m^6^A and fruit ripening. Namely, m^6^A presence in the coding sequence (CDS) appears to be ripening-specific and tends to stabilise the mRNAs, whilst m^6^A around the stop codons and within the 3’UTR is negatively correlated with the abundance of associated mRNA (Zhou et al. 2021). The same study found a notable upregulation of the RNA methyltransferase genes *MTA* and *MTB* during the onset of ripening, and that overexpression of *MTA* or *MTB* in strawberry lines led to an early fruit-ripening phenotype (Zhou et al. 2021). Moreover, the study showed that MTA- and MTB-guided m^6^A modification facilitated the ripening of strawberry fruit by either enhancing mRNA stability or promoting translation efficiency of genes in the ABA biosynthesis and signalling pathways (Zhou et al. 2021). In another study, apple (*Malus hupehensis*) m^6^A reader YTH family members were overexpressed in tomato which led to accelerated ripening in the transgenic lines (Wang et al. 2017). These findings demonstrate that m^6^A modification has an important role in Rosaceous plant development, particularly during fruit ripening.

RNA m^6^A modification has also been shown to be involved in leaf senescence in a study where the overexpression of the apple YTH family members showed accelerated leaf senescence in transgenic *Arabidopsis* and apple plants (Wang et al. 2017). m^6^A modification has also been implicated in grafting in apple, where a comprehensive epitranscriptomic-wide analysis showed that m^6^A hypermethylation was observed in heterografted, but not in self-grafted experimental combinations (Xu et al. 2023a).

The above studies show that recent progress in the investigation of plant m^6^A modification has reached an exciting stage and its relevance for a range of plant physiological processes in Rosaceous species is beginning to be revealed.

## Conclusions

This review summarises and discusses DNA and RNA methylation pathways and their dynamic involvement in Rosaceous developmental events. In the Rosaceae, most of the methylation maintenance and demethylation enzymes have been discovered and many of the major components of RdDM have also been reported. However, less information is currently available on the factors that provide cross-talk between RdDM and other chromatin modifications, and hence, future studies in this field should be able to reveal more about the interplay between different epigenetic mechanisms in the species of this family. Several recent studies have uncovered the involvement of 5mC DNA methylation in major developmental events of Rosaceous species, with roles in bud dormancy, plant regeneration and fruit ripening being particularly well documented. Whilst many of the studies on Rosaceae thus far have focused on global methylation patterns, future studies might be able to provide more information on key genomic regions and types of epigenetic regulation implicated in particular traits and developmental events, and therefore provide a more mechanistic approach that could be used for implementing epigenetic knowledge in breeding. In addition to unravelling the mechanisms and roles of 5 mC DNA methylation, there is a need to understand other, more recently discovered nucleic acid modifications and their biological roles. Whilst our understanding of the plant epitranscriptome is still in very early stages and will no doubt accelerate in the future, studies in Rosaceae have already yielded some of the m^6^A writer, reader and eraser components, and demonstrated important roles for RNA m^6^A modifications in fruit ripening and senescence processes.

## Data Availability

Data availability is not applicable to this publication as no new datasets were generated or analysed in the present study.

## References

[CR1] Aldwinckle H, Malnoy M (2009) Plant regeneration and transformation in the Rosaceae. Transgenic Plant J 3:1–38

[CR2] Arribas-Hernández L, Brodersen P (2020) Occurrence and functions of m6A and other covalent modifications in plant mRNA. Plant Physiol 182:79–96. 10.1104/pp.19.01156 31748418 10.1104/pp.19.01156 PMC6945878

[CR3] Bäurle I, Dean C (2006) The timing of developmental transitions in plants. Cell 125(4):655–664. 10.1016/j.cell.2006.05.005 16713560 10.1016/j.cell.2006.05.005

[CR4] Belal MA, Ezzat M, Zhang Y et al (2022) Integrative analysis of the DICER-like (DCL) genes from peach (*Prunus persica*): A critical role in response to drought stress. Front Ecol Evol 10:923166. 10.3389/fevo.2022.923166

[CR5] Bélanger S, Zhan J, Meyers BC (2022) Genome-wide analysis of small RNA biogenesis proteins refine the evolution of Dicer-like and Argonaute gene families in flowering plants. bioRxiv. 10.1101/2022.01.18.476847

[CR6] Bitonti MB, Cozza R, Chiappetta A et al (2002) Distinct nuclear organization, DNA methylation pattern and cytokinin distribution mark juvenile, juvenile-like and adult vegetative apical meristems in peach (Prunus persica (L.) Batsch). J Exp Bot 53(371):1047–1054. 10.1093/jexbot/53.371.1047 11971916 10.1093/jexbot/53.371.1047

[CR7] Burn JE, Bagnall DJ, Metzger JD et al (1993) DNA methylation, vernalization, and the initiation of flowering. Proc Natl Acad Sci USA 90(1):287–291. 10.1073/pnas.90.1.287 11607346 10.1073/pnas.90.1.287 PMC45645

[CR8] Canton M, Forestan C, Bonghi C, Varotto S (2021) Meta-analysis of RNA-Seq studies reveals genes with dominant functions during flower bud endo- to eco-dormancy transition in *Prunus* species. Sci Rep 11(1):13173. 10.1038/s41598-021-92600-6 34162991 10.1038/s41598-021-92600-6 PMC8222350

[CR9] Cao Q, Feng Y, Dai X, Huang L, Li J, Tao P, Crabbe MJC, Zhang T, Qiao Q (2021) Dynamic changes of DNA methylation during wild strawberry (*Fragaria nilgerrensis*) tissue culture. Front Plant Sci 12:765383. 10.3389/fpls.2021.765383 34917103 10.3389/fpls.2021.765383 PMC8669611

[CR10] Chen Y, Li D, Zhang X et al (2023) Azacytidine-induced hypomethylation delays senescence and coloration in harvested strawberries by stimulating antioxidant enzymes and modulating abscisate metabolism to minimize anthocyanin overproduction. Food Chem 407:135189. 10.1016/j.foodchem.2022.135189 36525805 10.1016/j.foodchem.2022.135189

[CR11] Cheng J, Niu Q, Zhang B et al (2018) Downregulation of RdDM during strawberry fruit ripening. Genome Biol 19(1):212. 10.1186/s13059-018-1587-x 30514401 10.1186/s13059-018-1587-x PMC6280534

[CR12] Cory H, Passarelli S, Szeto J et al (2018) The role of polyphenols in human health and food systems: A mini-review. Front Nutr 5:87. 10.3389/fnut.2018.00087 30298133 10.3389/fnut.2018.00087 PMC6160559

[CR13] D’Amico-Willman KM, Niederhuth CE, Willman MR et al (2021) Integrated analysis of the methylome and transcriptome of twin almonds (Prunus dulcis [Mill.] D.A. Webb) reveals genomic features associated with non-infectious bud failure. BioRxiv. 10.1101/2021.02.08.430330

[CR14] Dalakouras A, Vlachostergios D (2021) Epigenetic approaches to crop breeding: current status and perspectives. J Exp Bot 72(15):5356–5371. 10.1093/jxb/erab227 34017985 10.1093/jxb/erab227

[CR15] Di Lorenzo C, Colombo F, Biella S et al (2021) Polyphenols and human health: the role of bioavailability. Nutrients 13(1):273. 10.3390/nu13010273 33477894 10.3390/nu13010273 PMC7833401

[CR16] Eduardo I, Chietera G, Bassi D et al (2010) Identification of key odour volatile compounds in the essential oil of nine peach accessions. J Sci Food Agric 90(7):1146–1154. 10.1002/jsfa.3932 20393995 10.1002/jsfa.3932

[CR17] Fan W, Wang L, Lei Z et al (2022) Suppression of transposon mobilization by m^6^ A-mediated RNA sequestration in stress granules. BioRxiv. 10.1101/2022.03.22.48539836093346

[CR18] FAOSTAT Crops and livestock products. In: Food and Agriculture Organization of the United Nations. FAOSTAT Statistical Database. https://www.fao.org/faostat/en/#data/QCL/visualize. Accessed 5 Dec 2023

[CR19] Finnegan EJ, Genger RK, Kovac K et al (1998) DNA methylation and the promotion of flowering by vernalization. Proc Natl Acad Sci USA 95(10):5824–5829. 10.1073/pnas.95.10.5824 9576969 10.1073/pnas.95.10.5824 PMC20464

[CR20] Fresnedo-Ramírez J, Chan HM, Parfitt DE et al (2017) Genome-wide DNA-(de)methylation is associated with noninfectious bud-failure exhibition in almond (Prunus dulcis [Mill.] D.A. Webb). Sci Rep 7:42686. 10.1038/srep42686 28202904 10.1038/srep42686 PMC5311954

[CR21] Gao Z, Liu H-L, Daxinger L et al (2010) An RNA polymerase II- and AGO4-associated protein acts in RNA-directed DNA methylation. Nature 465:106–109. 10.1038/nature09025 20410883 10.1038/nature09025 PMC2865564

[CR22] Gehring M, Huh JH, Hsieh T-F et al (2006) DEMETER DNA glycosylase establishes MEDEA polycomb gene self-imprinting by allele-specific demethylation. Cell 124(3):495–506. 10.1016/j.cell.2005.12.034 16469697 10.1016/j.cell.2005.12.034 PMC4106368

[CR23] Giannino D, Mele G, Cozza R et al (2003) Isolation and characterization of a maintenance DNA-methyltransferase gene from peach (Prunus persica [L.] Batsch): transcript localization in vegetative and reproductive meristems of triple buds. J Exp Bot 54(393):2623–2633. 10.1093/jxb/erg292 14563834 10.1093/jxb/erg292

[CR24] Gong Z, Morales-Ruiz T, Ariza RR et al (2002) ROS1, a repressor of transcriptional gene silencing in *Arabidopsis*, encodes a DNA glycosylase/lyase. Cell 111(6):803–814. 10.1016/s0092-8674(02)01133-9 12526807 10.1016/s0092-8674(02)01133-9

[CR25] Gu T, Ren S, Wang Y et al (2016) Characterization of DNA methyltransferase and demethylase genes in *Fragaria vesca*. Mol Genet Genomics 291:1333–1345. 10.1007/s00438-016-1187-y 26956009 10.1007/s00438-016-1187-y

[CR26] Guo X, Ma Z, Zhang Z et al (2017) Small RNA-sequencing links physiological changes and RdDM process to vegetative-to-floral transition in apple. Front Plant Sci 8:873. 10.3389/fpls.2017.00873 28611800 10.3389/fpls.2017.00873 PMC5447065

[CR27] Guo T, Liu C, Meng F et al (2022) The m6 A reader MhYTP2 regulates MdMLO19 mRNA stability and antioxidant genes translation efficiency conferring powdery mildew resistance in apple. Plant Biotechnol J 20(3):511–525. 10.1111/pbi.13733 34679252 10.1111/pbi.13733 PMC8882777

[CR28] Hackett WP, Murray JR (1993) Maturation and rejuvenation in woody species. In: Ahuja MR (ed) Micropropagation of woody plants. Springer, Netherlands, Dordrecht, pp 93–105

[CR29] Han C, Dong H, Qiao Q et al (2023) Comparative genomic analysis of N6-methyladenosine regulators in nine rosaceae species and functional characterization in response to drought stress in pear. Horti Plant J 9(4):693–704. 10.1016/j.hpj.2022.09.008

[CR30] Harris CJ, Scheibe M, Wongpalee SP et al (2018) A DNA methylation reader complex that enhances gene transcription. Science 362(6419):1182–1186. 10.1126/science.aar7854 30523112 10.1126/science.aar7854 PMC6353633

[CR31] He L, Zhao C, Zhang Q et al (2021) Pathway conversion enables a double-lock mechanism to maintain DNA methylation and genome stability. Proc Natl Acad Sci USA 118(35):e2107320118. 10.1073/pnas.2107320118 34453006 10.1073/pnas.2107320118 PMC8536323

[CR32] Huijser P, Schmid M (2011) The control of developmental phase transitions in plants. Development 138(19):4117–4129. 10.1242/dev.063511 21896627 10.1242/dev.063511

[CR33] Iezzoni AF, Luby MJ, J, et al (2020) RosBREED: bridging the chasm between discovery and application to enable DNA-informed breeding in rosaceous crops. Hortic Res 7:177. 10.1038/s41438-020-00398-7 33328430 10.1038/s41438-020-00398-7 PMC7603521

[CR34] Ji L, Chen X (2012) Regulation of small RNA stability: methylation and beyond. Cell Res 22:624–636. 10.1038/cr.2012.36 22410795 10.1038/cr.2012.36 PMC3317568

[CR35] Jiang S, Wang N, Chen M et al (2020) Methylation of MdMYB1 locus mediated by RdDM pathway regulates anthocyanin biosynthesis in apple. Plant Biotechnol J 18:1736–1748. 10.1111/pbi.13337 31930634 10.1111/pbi.13337 PMC7336386

[CR36] Jiménez-Ramírez IA, Pijeira-Fernández G, Moreno-Cálix DM, De-la-Peña C (2022) Same modification, different location: the mythical role of N6-adenine methylation in plant genomes. Planta 256:9. 10.1007/s00425-022-03926-y 35696004 10.1007/s00425-022-03926-y

[CR37] Jing X, Xu L, Huai X et al (2023) Genome-wide identification and characterization of Argonaute, Dicer-like and RNA-dependent RNA polymerase gene families and their expression analyses in *Fragaria* spp. Genes 14(1):121. 10.3390/genes14010121 36672862 10.3390/genes14010121 PMC9859564

[CR38] Johnson LM, Du J, Hale CJ et al (2017) Corrigendum: SRA- and SET-domain-containing proteins link RNA polymerase V occupancy to DNA methylation. Nature 543:136. 10.1038/nature21398 28178234 10.1038/nature21398

[CR39] Kankel MW, Ramsey DE, Stokes TL et al (2003) *Arabidopsis* MET1 cytosine methyltransferase mutants. Genetics 163(3):1109–1122. 10.1093/genetics/163.3.1109 12663548 10.1093/genetics/163.3.1109 PMC1462485

[CR40] Kierzek E, Kierzek R (2003) The thermodynamic stability of RNA duplexes and hairpins containing N6-alkyladenosines and 2-methylthio-N6-alkyladenosines. Nucleic Acids Res 31(15):4472–4480. 10.1093/nar/gkg633 12888507 10.1093/nar/gkg633 PMC169893

[CR41] Kofler J, Milyaev A, Würtz B et al (2022) Proteomic differences in apple spur buds from high and non-cropping trees during floral initiation. J Proteomics 253:104459. 10.1016/j.jprot.2021.104459 34923173 10.1016/j.jprot.2021.104459

[CR42] Kumar G, Rattan UK, Singh AK (2016) Chilling-mediated DNA methylation changes during dormancy and its release reveal the importance of epigenetic regulation during winter dormancy in apple (Malus x domestica Borkh.). PLoS ONE 11(2):e0149934. 10.1371/journal.pone.0149934 26901339 10.1371/journal.pone.0149934 PMC4763039

[CR43] Lang GA, Early JD, Martin GC, Darnell RL (1987) Endo-, Para, and Ecodormancy: physiological terminology and classification for dormancy research. HortScience 22(5):701. 10.21273/HORTSCI.22.5.701b

[CR44] Lang Z, Wang Y, Tang K et al (2017) Critical roles of DNA demethylation in the activation of ripening-induced genes and inhibition of ripening-repressed genes in tomato fruit. Proc Natl Acad Sci USA 114(22):E4511–E4519. 10.1073/pnas.1705233114 28507144 10.1073/pnas.1705233114 PMC5465898

[CR45] Lang X, Yu C, Shen M et al (2023) PRMD: an integrated database for plant RNA modifications. Nucleic Acids Res 52(D1):D1597–D1613. 10.1093/nar/gkad851 10.1093/nar/gkad851 PMC10768107 37831097

[CR46] Lardon R, Geelen D (2020) Natural variation in plant pluripotency and regeneration. Plants 9(10):1261. 10.3390/plants9101261 32987766 10.3390/plants9101261 PMC7598583

[CR47] Law JA, Du J, Hale CJ et al (2013) Polymerase IV occupancy at RNA-directed DNA methylation sites requires SHH1. Nature 498:385–389. 10.1038/nature12178 23636332 10.1038/nature12178 PMC4119789

[CR48] Lee J, Jang H, Shin H et al (2014) AP endonucleases process 5-methylcytosine excision intermediates during active DNA demethylation in *Arabidopsis*. Nucleic Acids Res 42(18):11408–11418. 10.1093/nar/gku834 25228464 10.1093/nar/gku834 PMC4191409

[CR49] Lei M, Zhang H, Julian R et al (2015) Regulatory link between DNA methylation and active demethylation in *Arabidopsis*. Proc Natl Acad Sci USA 112(11):3553–3557. 10.1073/pnas.1502279112 25733903 10.1073/pnas.1502279112 PMC4371987

[CR50] Li W, Ma Y, Zheng C, Li G (2021) Variations of cytosine methylation patterns between staminate and perfect flowers within andromonoecious *Taihangia rupestris* (Rosaceae) revealed by methylation-sensitive amplification polymorphism. J Plant Growth Regul 41:351–363. 10.1007/s00344-021-10308-3

[CR51] Li Y, Shi Y, Li Y et al (2023) DNA methylation mediated by RdDM pathway and demethylation affects furanone accumulation through regulation of quinone oxidoreductase in strawberry. Hortic Res 10:uhad131. 10.1093/hr/uhad131 37560014 10.1093/hr/uhad131 PMC10407599

[CR52] Liang Z, Geng Y, Gu X (2018a) Adenine methylation: New epigenetic marker of DNA and mRNA. Mol Plant 11(10):1219–1221. 10.1016/j.molp.2018.08.001 30118810 10.1016/j.molp.2018.08.001

[CR53] Liang Z, Shen L, Cui X et al (2018b) DNA N6-Adenine methylation in *Arabidopsis thaliana*. Dev Cell 45(3):406-416.e3. 10.1016/j.devcel.2018.03.012 29656930 10.1016/j.devcel.2018.03.012

[CR54] Liang Z, Riaz A, Chachar S et al (2020) Epigenetic modifications of mRNA and DNA in plants. Mol Plant 13(1):14–30. 10.1016/j.molp.2019.12.007 31863849 10.1016/j.molp.2019.12.007

[CR55] Lindroth AM, Cao X, Jackson JP et al (2001) Requirement of CHROMOMETHYLASE3 for maintenance of CpXpG methylation. Science 292(5524):2077–2080. 10.1126/science.1059745 11349138 10.1126/science.1059745

[CR56] Liu C, Li H, Lin J et al (2018) Genome-wide characterization of DNA demethylase genes and their association with salt response in *Pyrus*. Genes 9(8):398. 10.3390/genes9080398 30082643 10.3390/genes9080398 PMC6116010

[CR57] Liu Z-Y, Xing J-F, Chen W et al (2019) MDR: an integrative DNA N6-methyladenine and N4-methylcytosine modification database for Rosaceae. Hortic Res 6:78. 10.1038/s41438-019-0160-4 31240103 10.1038/s41438-019-0160-4 PMC6572862

[CR58] Liu Z, Ma H, Jung S et al (2020) Developmental mechanisms of fleshy fruit diversity in Rosaceae. Annu Rev Plant Biol 71:547–573. 10.1146/annurev-arplant-111119-021700 32442388 10.1146/annurev-arplant-111119-021700

[CR59] Liu D, Mu Q, Li X et al (2022) The callus formation capacity of strawberry leaf explant is modulated by DNA methylation. Hortic Res 9:uhab073. 10.1093/hr/uhab073 35043170 10.1093/hr/uhab073 PMC8947209

[CR60] Ma C, Liang B, Chang B et al (2018) Transcriptome profiling reveals transcriptional regulation by DNA methyltransferase inhibitor 5-Aza-2’-deoxycytidine enhancing red pigmentation in bagged “granny smith” apples (*Malus domestica*). Int J Mol Sci 19(10):3133. 10.3390/ijms19103133 30322020 10.3390/ijms19103133 PMC6213223

[CR61] Mao X, Hou N, Liu Z, He J (2021) Profiling of N6-Methyladenosine (m6A) modification landscape in response to drought stress in apple (Malus prunifolia (Willd.) Borkh). Plants 11(1):103. 10.3390/plants11010103 35009106 10.3390/plants11010103 PMC8747461

[CR62] Martínez-Rivas FJ, Blanco-Portales R, Molina-Hidalgo FJ et al (2022) Azacytidine arrests ripening in cultivated strawberry (*Fragaria × ananassa*) by repressing key genes and altering hormone contents. BMC Plant Biol 22(1):278. 10.1186/s12870-022-03670-1 35672704 10.1186/s12870-022-03670-1 PMC9172142

[CR63] Matzke MA, Mosher RA (2014) Erratum: RNA-directed DNA methylation: an epigenetic pathway of increasing complexity. Nat Rev Genet 15:570. 10.1038/nrg3794 10.1038/nrg3683 24805120

[CR64] McCue AD, Panda K, Nuthikattu S et al (2015) Argonaute 6 bridges transposable element mRNA-derived siRNAs to the establishment of DNA methylation. EMBO J 34:20–35. 10.15252/embj.201489499 25388951 10.15252/embj.201489499 PMC4291478

[CR65] Mette MF, Aufsatz W, van der Winden J, Matzke MA, Matzke AJM (2000) Transcriptional silencing and promoter methylation triggered by double-stranded RNA. EMBO J 19:5194–5201. 10.1093/emboj/19.19.5194 11013221 10.1093/emboj/19.19.5194 PMC302106

[CR66] Miao Z, Zhang T, Xie B et al (2022) Evolutionary implications of the RNA N6-methyladenosine methylome in plants. Mol Biol Evol 39(1):msab299. 10.1093/molbev/msab299 34633447 10.1093/molbev/msab299 PMC8763109

[CR67] Mirzaei K, Bahramnejad B, Shamsifard MH, Zamani W (2014) *In silico* identification, phylogenetic and bioinformatic analysis of argonaute genes in plants. Int J Genomics 2014:967461. 10.1155/2014/967461 25309901 10.1155/2014/967461 PMC4181786

[CR68] Narváez G, Muñoz-Espinoza C, Soto E et al (2022) Global methylation analysis using MSAP reveals differences in chilling-associated dna methylation changes during dormancy release in contrasting sweet cherry varieties. Horticulturae 8(10):962. 10.3390/horticulturae8100962

[CR69] Nie F, Tang Q, Liu Y et al (2022) RNAME: A comprehensive database of RNA modification enzymes. Comput Struct Biotechnol J 20:6244–6249. 10.1016/j.csbj.2022.11.022 36420165 10.1016/j.csbj.2022.11.022 PMC9678767

[CR70] Nishat ZS, Hasan MdS, Islam MdS et al (2022) Identification of epitranscriptomic methylation marker genes in *Arabidopsis* and their expression profiling in response to developmental, anatomical, and environmental modulations. Current Plant Biology 30:100247. 10.1016/j.cpb.2022.100247

[CR71] Nuthikattu S, McCue AD, Panda K et al (2013) The initiation of epigenetic silencing of active transposable elements is triggered by RDR6 and 21–22 nucleotide small interfering RNAs. Plant Physiol 162(1):116–131. 10.1104/pp.113.216481 23542151 10.1104/pp.113.216481 PMC3641197

[CR72] Ogah O, Watkins CS, Ubi BE, Oraguzie NC (2014) Phenolic compounds in Rosaceae fruit and nut crops. J Agric Food Chem 62(39):9369–9386. 10.1021/jf501574q 25198667 10.1021/jf501574q

[CR73] Ortega-Galisteo AP, Morales-Ruiz T, Ariza RR, Roldán-Arjona T (2008) *Arabidopsis* DEMETER-LIKE proteins DML2 and DML3 are required for appropriate distribution of DNA methylation marks. Plant Mol Biol 67:671–681. 10.1007/s11103-008-9346-0 18493721 10.1007/s11103-008-9346-0

[CR74] Osorio S, Scossa F, Fernie AR (2013) Molecular regulation of fruit ripening. Front Plant Sci 4:198. 10.3389/fpls.2013.00198 23785378 10.3389/fpls.2013.00198 PMC3682129

[CR75] Pan C, Sretenovic S, Qi Y (2021) CRISPR/dCas-mediated transcriptional and epigenetic regulation in plants. Curr Opin Plant Biol 60:101980. 10.1016/j.pbi.2020.101980 33401227 10.1016/j.pbi.2020.101980

[CR76] Pappalardo XG, Barra V (2021) Losing DNA methylation at repetitive elements and breaking bad. Epigenet Chromatin 14:25. 10.1186/s13072-021-00400-z 10.1186/s13072-021-00400-z PMC8173753 34082816

[CR77] Peace CP (2017) DNA-informed breeding of Rosaceous crops: promises, progress and prospects. Hortic Res 4:17006. 10.1038/hortres.2017.6 28326185 10.1038/hortres.2017.6 PMC5350264

[CR78] Penterman J, Zilberman D, Huh JH et al (2007) DNA demethylation in the *Arabidopsis* genome. Proc Natl Acad Sci USA 104(16):6752–6757. 10.1073/pnas.0701861104 17409185 10.1073/pnas.0701861104 PMC1847597

[CR79] Pérez-Llorca M, Munné-Bosch S (2021) Aging, stress, and senescence in plants: what can biological diversity teach us? Geroscience 43:167–180. 10.1007/s11357-021-00336-y 33590435 10.1007/s11357-021-00336-y PMC8050193

[CR80] Pontier D, Picart C, El Baidouri M et al (2019) The m6A pathway protects the transcriptome integrity by restricting RNA chimera formation in plants. Life Sci Alliance 2(3):e201900393. 10.26508/lsa.201900393 31142640 10.26508/lsa.201900393 PMC6545605

[CR81] Qian M, Sun Y, Allan AC et al (2014) The red sport of “Zaosu” pear and its red-striped pigmentation pattern are associated with demethylation of the PyMYB10 promoter. Phytochemistry 107:16–23. 10.1016/j.phytochem.2014.08.001 25168359 10.1016/j.phytochem.2014.08.001

[CR82] Rothkegel K, Sánchez E, Montes C et al (2017) DNA methylation and small interference RNAs participate in the regulation of MADS-box genes involved in dormancy in sweet cherry (Prunus avium L.). Tree Physiol 37(12):1739–1751. 10.1093/treephys/tpx055 28541567 10.1093/treephys/tpx055

[CR83] Rothkegel K, Sandoval P, Soto E et al (2020) Dormant but active: Chilling accumulation modulates the epigenome and transcriptome of *Prunus avium* during bud dormancy. Front Plant Sci 11:1115. 10.3389/fpls.2020.01115 32765576 10.3389/fpls.2020.01115 PMC7380246

[CR84] Rothkegel K, Espinoza A, Sanhueza D et al (2021) Identification of DNA methylation and transcriptomic profiles associated with fruit mealiness in Prunus persica (L.) Batsch. Front Plant Sci 12:684130. 10.3389/fpls.2021.684130 34178003 10.3389/fpls.2021.684130 PMC8222998

[CR85] Růžička K, Zhang M, Campilho A et al (2017) Identification of factors required for m6 A mRNA methylation in *Arabidopsis* reveals a role for the conserved E3 ubiquitin ligase HAKAI. New Phytol 215(1):157–172. 10.1111/nph.14586 28503769 10.1111/nph.14586 PMC5488176

[CR86] Satayaki PRV, Gehring M (2017) DNA methylation and imprinting in plants: machinery and mechanisms. Crit Rev Biochem Mol Biol 52:163–175. 10.1080/10409238.2017.1279119 28118754 10.1080/10409238.2017.1279119

[CR87] Schibler U, Kelley DE, Perry RP (1977) Comparison of methylated sequences in messenger RNA and heterogeneous nuclear RNA from mouse L cells. J Mol Biol 115(4):695–714. 10.1016/0022-2836(77)90110-3 592376 10.1016/0022-2836(77)90110-3

[CR88] Serra S, Anthony B, Masia A et al (2020) Determination of biochemical composition in peach (Prunus persica L. Batsch) accessions characterized by different flesh color and textural typologies. Foods 9(10):1452. 10.3390/foods9101452 33066145 10.3390/foods9101452 PMC7601976

[CR89] Shibuya K, Fukushima S, Takatsuji H (2009) RNA-directed DNA methylation induces transcriptional activation in plants. Proc Natl Acad Sci USA 106(5):1660–1665. 10.1073/pnas.0809294106 19164525 10.1073/pnas.0809294106 PMC2629447

[CR90] Shulaev V, Korban SS, Sosinski B et al (2008) Multiple models for Rosaceae genomics. Plant Physiol 147(3):985–1003. 10.1104/pp.107.115618 18487361 10.1104/pp.107.115618 PMC2442536

[CR91] Stroud H, Do T, Du J et al (2014) Non-CG methylation patterns shape the epigenetic landscape in *Arabidopsis*. Nat Struct Mol Biol 21:64–72. 10.1038/nsmb.2735 24336224 10.1038/nsmb.2735 PMC4103798

[CR92] Telias A, Lin-Wang K, Stevenson DE et al (2011) Apple skin patterning is associated with differential expression of MYB10. BMC Plant Biol 11:93. 10.1186/1471-2229-11-93 21599973 10.1186/1471-2229-11-93 PMC3127826

[CR93] Theler D, Dominguez C, Blatter M et al (2014) Solution structure of the YTH domain in complex with N6-methyladenosine RNA: a reader of methylated RNA. Nucleic Acids Res 42(22):13911–13919. 10.1093/nar/gku1116 25389274 10.1093/nar/gku1116 PMC4267619

[CR94] Varotto S, Tani E, Abraham E et al (2020) Epigenetics: possible applications in climate-smart crop breeding. J Exp Bot 71(17):5223–5236. 10.1093/jxb/eraa188 32279074 10.1093/jxb/eraa188 PMC7475248

[CR95] Wang Z, Meng D, Wang A et al (2013) The methylation of the PcMYB10 promoter is associated with green-skinned sport in Max Red Bartlett pear. Plant Physiol 162(2):885–896. 10.1104/pp.113.214700 23629835 10.1104/pp.113.214700 PMC3668077

[CR96] Wang N, Guo T, Wang P et al (2017) Functional analysis of apple MhYTP1 and MhYTP2 genes in leaf senescence and fruit ripening. Sci Hortic 221:23–32. 10.1016/j.scienta.2017.04.018

[CR97] Wang L, Tang T, Wang W et al (2021) Multi-Omics landscape of DNA methylation regulates browning in “Fuji” apple. Front Nutr 8:800489. 10.3389/fnut.2021.800489 35198585 10.3389/fnut.2021.800489 PMC8859415

[CR98] Wei L-H, Song P, Wang Y et al (2018) The m6A reader ECT2 controls trichome morphology by affecting mrna stability in *Arabidopsis*. Plant Cell 30(5):968–985. 10.1105/tpc.17.00934 29716990 10.1105/tpc.17.00934 PMC6002187

[CR99] Wei C, Liu H, Cao X et al (2021) Synthesis of flavour-related linalool is regulated by PpbHLH1 and associated with changes in DNA methylation during peach fruit ripening. Plant Biotechnol J 19:2082–2096. 10.1111/pbi.13638 34036730 10.1111/pbi.13638 PMC8486240

[CR100] Wiener D, Schwartz S (2021) The epitranscriptome beyond m6A. Nat Rev Genet 22:119–131. 10.1038/s41576-020-00295-8 33188361 10.1038/s41576-020-00295-8

[CR101] Xie S-Q, Xing J-F, Zhang X-M et al (2019) N6-Methyladenine DNA modification in the woodland strawberry (*Fragaria vesca*) genome reveals a positive relationship with gene transcription. Front Genet 10:1288. 10.3389/fgene.2019.01288 31998359 10.3389/fgene.2019.01288 PMC6967393

[CR102] Xing L, Li Y, Qi S et al (2019) Comparative RNA-sequencing and DNA methylation analyses of apple (Malus domestica Borkh.) buds with diverse flowering capabilities reveal novel insights into the regulatory mechanisms of flower bud formation. Plant Cell Physiol 60(8):1702–1721. 10.1093/pcp/pcz080 31077318 10.1093/pcp/pcz080

[CR103] Xing L, Qi S, Zhou H et al (2020) Epigenomic regulatory mechanism in vegetative phase transition of *Malus hupehensis*. J Agric Food Chem 68(17):4812–4829. 10.1021/acs.jafc.0c00478 32227940 10.1021/acs.jafc.0c00478

[CR104] Xing Y, Xie Z, Sun W et al (2021) The RNA directed DNA Methylation (RdDM) Pathway regulates anthocyanin biosynthesis in crabapple (Malus cv. Spp.) leaves by methylating the McCOP1 promoter. Plants 10(11):2466. 10.3390/plants10112466 34834829 10.3390/plants10112466 PMC8618851

[CR105] Xu R, Liu C, Li N, Zhang S (2016) Global identification and expression analysis of stress-responsive genes of the Argonaute family in apple. Mol Genet Genomics 291(6):2015–2030. 10.1007/s00438-016-1236-6 27475441 10.1007/s00438-016-1236-6

[CR106] Xu J, Zhou S, Gong X et al (2018) Single-base methylome analysis reveals dynamic epigenomic differences associated with water deficit in apple. Plant Biotechnol J 16:672–687. 10.1111/pbi.12820 28796917 10.1111/pbi.12820 PMC5787839

[CR107] Xu J, He J, Hu B et al (2023a) Global hypermethylation of the N6-methyladenosine RNA modification associated with apple heterografting. Plant Physiol 193(4):2513–2537. 10.1093/plphys/kiad470 37648253 10.1093/plphys/kiad470 PMC10663141

[CR108] Xu P, Li X, Fan J et al (2023b) Comprehensive Identification and Expression Analysis of the YTH Family of RNA-Binding Proteins in Strawberry. Plants 12(7):1449. 10.3390/plants12071449 37050075 10.3390/plants12071449 PMC10097400

[CR109] Yang Y, Tang K, Datsenka TU et al (2019) Critical function of DNA methyltransferase 1 in tomato development and regulation of the DNA methylome and transcriptome. J Integr Plant Biol 61(12):1224–1242. 10.1111/jipb.12778 30652405 10.1111/jipb.12778

[CR110] Yu L, Sun Y, Zhang X et al (2022) ROS1 promotes low temperature-induced anthocyanin accumulation in apple by demethylating the promoter of anthocyanin-associated genes. Hortic Res 9:uhac007. 10.1093/hr/uhac007 35147161 10.1093/hr/uhac007 PMC9123231

[CR111] Zaccara S, Ries RJ, Jaffrey SR (2023) Publisher Correction: Reading, writing and erasing mRNA methylation. Nat Rev Mol Cell Biol 24:770. 10.1038/s41580-023-00654-3 37604996 10.1038/s41580-023-00654-3

[CR112] Zemach A, Grafi G (2003) Characterization of *Arabidopsis thaliana* methyl-CpG-binding domain (MBD) proteins. Plant J 34(5):565–572. 10.1046/j.1365-313x.2003.01756.x 12787239 10.1046/j.1365-313x.2003.01756.x

[CR113] Zemach A, Kim MY, Hsieh P-H et al (2013) The *Arabidopsis* nucleosome remodeler DDM1 allows DNA methyltransferases to access H1-containing heterochromatin. Cell 153(1):193–205. 10.1016/j.cell.2013.02.033 23540698 10.1016/j.cell.2013.02.033 PMC4035305

[CR114] Zeng W, Huang H, Lin X et al (2021) Roles of DEMETER in regulating DNA methylation in vegetative tissues and pathogen resistance. J Integr Plant Biol 63(4):691–706. 10.1111/jipb.13037 33236824 10.1111/jipb.13037 PMC8251943

[CR115] Zhang L, Wang Y, Zhang X et al (2012) Dynamics of phytohormone and DNA methylation patterns changes during dormancy induction in strawberry (*Fragaria × ananassa* Duch.). Plant Cell Rep 31:155–165. 10.1007/s00299-011-1149-0 21935696 10.1007/s00299-011-1149-0

[CR116] Zhang H, Ma Z-Y, Zeng L et al (2013) DTF1 is a core component of RNA-directed DNA methylation and may assist in the recruitment of Pol IV. Proc Natl Acad Sci USA 110(20):8290–8295. 10.1073/pnas.1300585110 23637343 10.1073/pnas.1300585110 PMC3657815

[CR117] Zhang H, Tang K, Qian W et al (2014) An Rrp6-like protein positively regulates noncoding RNA levels and DNA methylation in *Arabidopsis*. Mol Cell 54(3):418–430. 10.1016/j.molcel.2014.03.019 24726328 10.1016/j.molcel.2014.03.019 PMC4023806

[CR118] Zhang H, Lang Z, Zhu J-K (2018a) Dynamics and function of DNA methylation in plants. Nat Rev Mol Cell Biol 19:489–506. 10.1038/s41580-018-0016-z 29784956 10.1038/s41580-018-0016-z

[CR119] Zhang Q, Liang Z, Cui X et al (2018b) N6-methyladenine DNA methylation in Japonica and Indica rice genomes and its association with gene expression, plant development, and stress responses. Mol Plant 11(12):1492–1508. 10.1016/j.molp.2018.11.005 30448535 10.1016/j.molp.2018.11.005

[CR120] Zhang Y, Bozorov TA, Li DX et al (2020) An efficient in vitro regeneration system from different wild apple (*Malus sieversii)* explants. Plant Methods 16:56. 10.1186/s13007-020-00599-0 32336979 10.1186/s13007-020-00599-0 PMC7175559

[CR121] Zhang G, Diao S, Song Y et al (2022) Genome-wide DNA N6-adenine methylation in sea buckthorn (Hippophae rhamnoides L.) fruit development. Tree Physiol 42(6):1286–1295. 10.1093/treephys/tpab177 34986489 10.1093/treephys/tpab177

[CR122] Zheng B, Liu J, Gao A et al (2022) Epigenetic reprogramming of H3K27me3 and DNA methylation during leaf-to-callus transition in peach. Hortic Res 9:uhac132. 10.1093/hr/uhac132 35937864 10.1093/hr/uhac132 PMC9350832

[CR123] Zheng G, Hu S, Cheng S et al (2023) Factor of DNA methylation 1 affects woodland strawberry plant stature and organ size via DNA methylation. Plant Physiol 191(1):335–351. 10.1093/plphys/kiac462 36200851 10.1093/plphys/kiac462 PMC9806633

[CR124] Zhong S, Li H, Bodi Z et al (2008) MTA is an *Arabidopsis* messenger RNA adenosine methylase and interacts with a homolog of a sex-specific splicing factor. Plant Cell 20(5):1278–1288. 10.1105/tpc.108.058883 18505803 10.1105/tpc.108.058883 PMC2438467

[CR125] Zhong X, Du J, Hale CJ et al (2014) Molecular mechanism of action of plant DRM de novo DNA methyltransferases. Cell 157(5):1050–1060. 10.1016/j.cell.2014.03.056 24855943 10.1016/j.cell.2014.03.056 PMC4123750

[CR126] Zhou C, Wang C, Liu H et al (2018) Identification and analysis of adenine N6-methylation sites in the rice genome. Nat Plants 4:554–563. 10.1038/s41477-018-0214-x 30061746 10.1038/s41477-018-0214-x

[CR127] Zhou L, Tian S, Qin G (2019) RNA methylomes reveal the m6A-mediated regulation of DNA demethylase gene SlDML2 in tomato fruit ripening. Genome Biol 20:156. 10.1186/s13059-019-1771-7 31387610 10.1186/s13059-019-1771-7 PMC6683476

[CR128] Zhou L, Tang R, Li X et al (2021) N6-methyladenosine RNA modification regulates strawberry fruit ripening in an ABA-dependent manner. Genome Biol 22:168. 10.1186/s13059-021-02385-0 34078442 10.1186/s13059-021-02385-0 PMC8173835

[CR129] Zhu H, Chen P-Y, Zhong S et al (2020a) Thermal-responsive genetic and epigenetic regulation of DAM cluster controlling dormancy and chilling requirement in peach floral buds. Hortic Res 7:114. 10.1038/s41438-020-0336-y 32821397 10.1038/s41438-020-0336-y PMC7395172

[CR130] Zhu Y-C, Zhang B, Allan AC et al (2020b) DNA demethylation is involved in the regulation of temperature-dependent anthocyanin accumulation in peach. Plant J 102(5):965–976. 10.1111/tpj.14680 31923329 10.1111/tpj.14680

